# Rhabdomyolysis Reported in Association with SARS-CoV-2 Infection and COVID-19 Vaccination: A Systematic Review

**DOI:** 10.3390/medicina62091769

**Published:** 2026-09-15

**Authors:** Fernando M. Runzer-Colmenares, Nelson Luis Cahuapaza-Gutierrez, Cielo Cinthya Calderon-Hernandez, Mariam Miyanay Umeres-Bravo, Ruth Angélica Rojas-De la Cruz, Tatiana Vanessa Villavicencio-Escudero, Renzo Pajuelo-Vasquez, Nathaly Enciso

**Affiliations:** 1 CHANGE Research Working Group, Universidad Científica del Sur, Lima 15842, Peru; 100065659@cientifica.edu.pe (N.L.C.-G.); 100052073@cientifica.edu.pe (C.C.C.-H.);; 2 Grupo de Medicina Regenerativa, Universidad Científica del Sur, Lima 150142, Peru; 3 Research Department, N y C—Center of Research and Medical Excellence (CRME), Lima 15067, Peru; 4 Facultad de Ciencias de la Salud, Carrera de Medicina, Universidad de Aquino Bolivia, Cochabamba, Bolivia

**Keywords:** rhabdomyolysis, SARS-CoV-2, COVID-19 vaccines, SARS-CoV-2 vaccines, systematic review (source: Medical Subjects Headings (MeSH) of National Library of Medicine (NLM))

## Abstract

*Background and Objectives*: The association between SARS-CoV-2 infection, COVID-19 vaccination, and the development of rhabdomyolysis remains unclear. This systematic review aimed to comprehensively synthesize and analyze the available evidence on the occurrence of rhabdomyolysis in the context of SARS-CoV-2 infection and COVID-19 vaccination. *Materials and Methods*: A literature search was conducted in PubMed, Scopus, EMBASE, and Web of Science. The review followed PRISMA 2020 guidelines, assessing methodological quality. Statistical analyses were performed using STATA v18. *Results*: A total of 133 patients with rhabdomyolysis associated with SARS-CoV-2 infection were identified from 93 case reports and 7 case series; the median age was 45.5 years (IQR: 24–62), with a predominance of males (72.9%). Additionally, 13 observational studies comprising 818 patients with rhabdomyolysis associated with SARS-CoV-2 infection were included. Among cases associated with SARS-CoV-2 vaccination, 20 patients were identified from 20 case reports; the median age was 52.5 years (IQR: 22.5–76.5), with a predominance of males (75%). These cases were primarily associated with mRNA-based vaccines, particularly Pfizer-BioNTech BNT162b2 (50%) and Moderna (20%), and occurred predominantly after the first and second doses. The most frequent clinical manifestations in both groups were fever, myalgia, and dark urine. Clinical outcomes were favorable in most patients, both among infection-associated cases (72.2%) and vaccination-associated cases (80%). *Conclusions*: Rhabdomyolysis has been documented during SARS-CoV-2 infection, although its true frequency remains uncertain, whereas cases following COVID-19 vaccination do not establish causality or platform-specific risks.

## 1. Introduction

Coronavirus disease 2019 (COVID-19), the cause of the recent pandemic, is a human upper respiratory tract infection caused by Severe Acute Respiratory Syndrome Coronavirus 2 (SARS-CoV-2) [1]. This virus binds via its spike glycoprotein to receptors on its host, with angiotensin-converting enzyme 2 (ACE2) as its main receptor [1]. COVID-19 has generated high morbidity and mortality worldwide, affecting all ages [2]. According to data from the World Health Organization (WHO), as of 15 March 2026, more than 779 million confirmed cases of COVID-19 and over 7 million deaths have been reported worldwide [3]. Vaccination is one of the most important preventive measures to contain the spread of COVID-19 [4]. FDA-approved COVID-19 vaccines include inactivated, viral vector, nucleic acid, and subunit vaccines [4]. These vaccines have demonstrated safety and clinical efficacy; however, the emergence of new SARS-CoV-2 variants may reduce the efficacy of these vaccines [5].

Rhabdomyolysis is a pathological syndrome characterized by the release of intracellular components into the blood following degradation and necrosis of skeletal muscle tissue [6]. The clinical manifestations of rhabdomyolysis vary from asymptomatic to clinical presentation characterized by myalgia and weakness associated with elevated creatine kinase, and in severe rhabdomyolysis, acute kidney injury may develop, increasing mortality by up to 80% [6,7]. The causes of this entity are diverse, vary according to age and geographic variation, and can be classified as acquired and genetic [6,7]. Acquired causes of rhabdomyolysis include trauma, hypoxic injuries, infections, hyperthermia, drugs, and toxins; on the other hand, genetic causes can be enzyme deficiencies and myopathies [7]. Each etiology of rhabdomyolysis through the oxidative stress generated leads to cell membrane damage, hypoxia, ATP depletion, release of degradative enzymes and free radicals; these mechanisms cause increased myofibril contraction and an inflammatory cascade resulting in skeletal muscle cell death [7].

Although SARS-CoV-2 primarily affects the respiratory system, it can also cause extrapulmonary effects by compromising various organs and systems, largely due to the presence of the ACE2 receptor [8]. Extrapulmonary manifestations associated with SARS-CoV-2 infection include thrombotic complications, cardiovascular complications, acute kidney injury, gastrointestinal complications, hyperglycemia and ketosis, neurological disorders, ocular symptoms, and dermatological complications [9]. On the other hand, COVID-19 vaccines can cause various adverse effects, some of which are mild, such as pain, redness, or swelling at the injection site. Other common adverse effects include fever, fatigue, headache, myalgia, nausea, vomiting, pruritus, chills, and arthralgia, and in some cases, even anaphylactic shock [10]. Among the rare adverse effects, cases of Guillain-Barré syndrome, deep vein thrombosis, pulmonary thromboembolism, nephrotic syndrome, acute kidney injury, and vesiculobullous skin reactions have been reported [11].

Rhabdomyolysis has been associated with SARS-CoV-2 infection as a potentially life-threatening complication, particularly in hospitalized patients [12]. Additionally, cases of rhabdomyolysis have been reported in the context of COVID-19 vaccination, suggesting a possible adverse effect [13]. However, these studies are limited to general descriptions, and the exact mechanisms underlying this association remain unclear [14]. Therefore, the primary objective of this study was to synthesize and analyze the available evidence on rhabdomyolysis cases associated with both SARS-CoV-2 infection and COVID-19 vaccination.

## 2. Materials and Methods

### 2.1. Protocol and Registration

The study protocol was reported in accordance with the “Preferred Reporting Items for Systematic Reviews and Meta-Analyses for Protocols” (PRISMA-P 2015) statement [15]. The review protocol was registered in the “International Prospective Register of Systematic Reviews” (PROSPERO) under registration number CRD42024627644 [16]. This study was conducted and reported following the PRISMA 2020 guidelines [17].

### 2.2. Eligibility Criteria

The eligibility criteria were based on the previously formulated PICO research question. The research question was formulated according to the key elements of the PICO framework: P (population), I/E (intervention/exposure), C (comparator), and O (outcomes). The population comprised all patients who developed new-onset rhabdomyolysis associated with either SARS-CoV-2 infection or COVID-19 vaccination. Rhabdomyolysis was defined as cases meeting one or more of the following criteria: (1) laboratory evidence of elevated creatine kinase (CK) levels > 1000 U/L or ≥3 times the upper limit of normal (ULN; 225 U/L); and/or (2) evidence of acute kidney injury (AKI) attributable to rhabdomyolysis, including myoglobinuria or an increase in serum creatinine of ≥0.3 mg/dL within 48 h or ≥50% within seven days. The interventions included all COVID-19 vaccines, regardless of vaccine platform (inactivated, viral vector, nucleic acid-based, or protein subunit vaccines) and vaccine dose (1st, 2nd, 3rd, or booster dose). The exposure of interest was COVID-19 resulting from SARS-CoV-2 infection. Comparators were not evaluated due to the nature of the studies included. The primary outcomes were clinical and demographic characteristics, diagnostic findings, management strategies, and clinical outcomes. Clinical outcomes were categorized as favorable, unfavorable, or death. A favorable outcome was defined as clinical improvement or recovery; an unfavorable outcome was defined as persistent symptoms, the occurrence of complications, or failure to document complete recovery in the primary study; and death was defined as the patient’s death during the reported follow-up period. The study included case reports, case series, and observational studies. The study excluded letters, comments, notes, correspondence, short communications, brief reports, narrative reviews, systematic reviews, meta-analyses, in vivo studies, in vitro studies, books, book chapters, news articles, and opinion pieces. Additionally, studies reporting other outcomes and those written in languages other than English were excluded.

### 2.3. Information Sources

The preliminary search was conducted on 20 March 2024 and was updated on 30 December 2024. The final search presented in this study was performed on 24 December 2025. A selective literature search was conducted in the following electronic databases: PubMed, Scopus, and EMBASE. Additionally, the Web of Science (WOS) platform was consulted. To ensure comprehensive search, manual screening of the reference lists of included studies was performed.

### 2.4. Search Strategy

A search strategy was designed using terms from Medical Subject Headings (MeSH) of the National Library of Medicine (NLM): “COVID-19 Vaccine,” “COVID-19,” and “Rhabdomyolysis,” combined with the Boolean operators AND and OR. The search was limited to the English language, but no restrictions were applied regarding the publication date. Full details of the search strategy for each database can be found in Supplementary Material S1.

### 2.5. Study Selection Process

One author downloaded all records into an EndNote X9 document to remove duplicates. The author then exported all references to Rayyan QCRI (https://www.rayyan.ai/, accessed on 10 January 2026). Two authors (CCCH and MMUB) independently screened the studies by title and abstract for potential inclusion. Subsequently, full-text reviews were conducted to determine eligibility. Any disagreements were resolved by a third author (NLCG).

### 2.6. Data Extraction Process

The data of interest were independently extracted by three reviewers and recorded in a Microsoft Word sheet previously prepared by the authors. The extracted data were subsequently compared to identify and resolve any discrepancies. Study investigators were not contacted regarding missing data due to the large number of included studies. The most relevant characteristics of the studies evaluating the association between rhabdomyolysis and SARS-CoV-2 infection were collected, including: first author’s name, year of publication, country, study type, medical history and comorbidities, age, sex, confirmation of SARS-CoV-2 infection, estimated time of rhabdomyolysis onset after infection, clinical manifestations, diagnosis, treatment, and disease progression.

For studies analyzing the association between rhabdomyolysis and COVID-19 vaccination, the following data were added: vaccine type, number of doses received, and time elapsed until symptom onset after administration. For the observational studies, the following data were extracted: first author, year of publication, country, study design, study period, age group, mean age, sex distribution, total sample size, number of cases, creatine kinase levels, percentage of acute kidney injury, frequency measures, and main findings.

### 2.7. Risk of Bias and Quality Assessment of Individual Studies

The risk of bias and quality of individual studies were assessed using the Joanna Briggs Institute (JBI) critical appraisal checklist for case reports and case series studies, calculating the total score for each study [18]. For the evaluation, the Newcastle-Ottawa Scale (NOS) was used for observational studies, including non-randomized studies, cohort studies, and case–control studies. The NOS assesses three domains: Selection, Comparability, and the determination of Exposure or Outcomes, for case–control and cohort studies, respectively [19]. Articles with a score ≥ 7 were considered low risk of bias, a score between 4 and 6 was considered high risk of bias, and a score < 4 was considered very high risk of bias. Three authors (NLCG, CCCH, and MMUB) independently performed the risk of bias and quality assessments. Any disagreements were resolved through mutual discussion.

### 2.8. Statistical Analysis (Data Synthesis)

STATA version 18 statistical software (StataCorporation, College Station, TX, USA) was used for data synthesis and analysis. Categorical variables were presented as absolute frequencies and proportions, *n* (%). Given that the data were mostly derived from case reports, an asymmetric distribution was assumed, and continuous variables were described using the median and interquartile range (IQR).

## 3. Results

### 3.1. Selection of Studies

**Figure 1:**
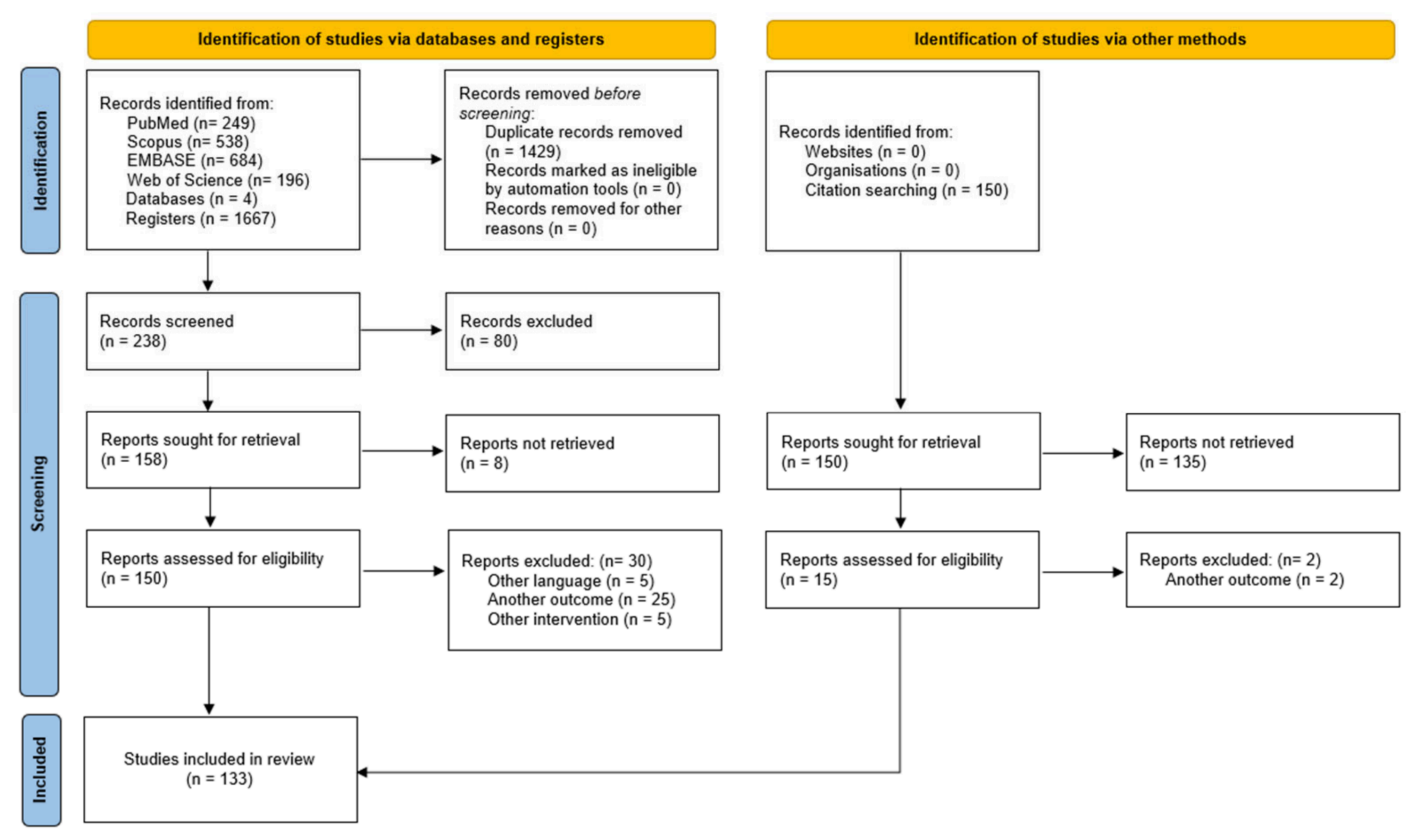
Selected studies on Rhabdomyolysis, SARS-CoV-2 infection and COVID-19 vaccination. Flow diagram “Preferred Reporting Items for Systematic Reviews and Meta-Analyses” (PRISMA-2020).

A selective literature search was conducted in four electronic databases: PubMed, Scopus, EMBASE, and Web of Science, identifying a total of 1667 records. After removing duplicates, 238 manuscripts remained. Subsequently, selection by title and abstract was performed, yielding 158 studies. Eight of these were excluded due to incomplete data. Then, 150 studies were assessed in full text, of which 120 records were included. The excluded studies used a different type of language, outcomes, or interventions than those established in the inclusion criteria. In addition, a manual search was carried out in the reference lists of the included studies, identifying 13 additional studies. In total, the present work included 133 manuscripts: 113 case reports, 7 case series, and 13 observational studies. Figure 1 provides a detailed overview of the study selection process.

### 3.2. Rhabdomyolysis and SARS-CoV-2

A total of 93 case reports and 7 case series describing the development of rhabdomyolysis associated with SARS-CoV-2 infection were included, published between 2020 and 2024. The frequency and proportion analysis by country showed that the countries with the largest number of reported cases were the United States (*n* = 76; 57.1%), Taiwan (*n* = 8; 6.0%), Japan (*n* = 7; 5.3%), and Iran (*n* = 5; 3.8%). When analyzed by continent, cases were reported primarily in North America (*n* = 78; 58.6%) and Asia (*n* = 31; 23.3%), followed by Europe (*n* = 14; 10.5%), South America (*n* = 6; 4.5%), Africa (*n* = 3; 2.3%), and Oceania (*n* = 1; 0.8%). Detailed characteristics of the included studies are provided in Supplementary Material Table S1.

#### Characteristics of the Included Studies

A total of 133 patients were reported in the studies analyzed. The median age was 45.5 years (IQR: 24–62). There was a predominance of males (72.9%) compared with females (26.3%), with an estimated ratio of 2.7:1. All patients tested positive for COVID-19. The most common clinical manifestations were fever (54.1%), dyspnea (41.4%), myalgia (38.3%), and cough (36.8%). Notably, 27.1% of patients had no relevant medical history. The most frequent comorbidities included hypertension (32.3%), diabetes mellitus (17.3%), and obesity (11.3%). In most cases, the diagnosis was established based on clinical and laboratory criteria. Elevated creatine kinase (CK) levels were observed in 96.9% of cases, followed by increased Aspartate Aminotransferase (AST) (60.9%), creatinine (39.1%), and Alanine Aminotransferase (ALT) (9.0%). Among complications, the most frequent was acute kidney injury (AKI) (16.5%), followed by cardiovascular events such as myocarditis, arrhythmias, cardiorespiratory arrest, and cardiac tamponade (7.5%). Treatment primarily consisted of fluid therapy (45.1%) and corticosteroid administration (25.6%). Clinical outcomes were favorable in most patients (72.2%); however, 31 deaths (23.3%) were reported, mainly related to complications. Table 1 provides a detailed description of the clinical and demographic characteristics of patients who developed new-onset rhabdomyolysis associated with SARS-CoV-2 infection.

### 3.3. Incidence and Prevalence of Rhabdomyolysis Associated with SARS-CoV-2 Infection

#### Characteristics of Studies Included

A total of 13 observational studies were included, of which 12 were cohort studies and 1 was a cross-sectional study. Collectively, these studies reported 818 cases of rhabdomyolysis among 7766 patients with COVID-19. Sample sizes ranged from 101 to 1079 patients, while the number of rhabdomyolysis cases ranged from 5 to 189. Several observational studies reported that rhabdomyolysis was more frequently observed in patients with more severe forms of COVID-19, particularly those requiring hospitalization or admission to intensive care units. Likewise, some studies reported an association between rhabdomyolysis and acute kidney injury, while others observed higher mortality among patients who developed rhabdomyolysis. However, these findings should be interpreted as observational associations and do not demonstrate that rhabdomyolysis independently contributes to COVID-19 severity, AKI, or mortality, given the potential influence of disease severity, comorbidities, and other confounding factors. Table 2 provides a detailed overview of the main characteristics of the included observational studies. The definitions of rhabdomyolysis and the methods used to identify or diagnose this condition in each study are presented in Supplementary Table S2.

### 3.4. Rhabdomyolysis and COVID-19 Vaccines

The development of rhabdomyolysis associated with vaccination was reported in 20 case report studies published between 2021 and 2025. The frequency–proportion analysis by country showed that most cases were reported in the USA (25%). By continent, the distribution was as follows: Europe (*n* = 9, 45%), North America (*n* = 6, 30%), and Asia (*n* = 5, 25%). Detailed characteristics of the included studies are presented in Supplementary Material S4.

#### Clinical and Demographic Characteristics

A total of 20 patients who developed rhabdomyolysis following COVID-19 vaccination were reported. A predominance of males was observed (75%). The median age was 52.5 years (IQR: 22.5–76.5). mRNA-based vaccines, including Pfizer-BioNTech BNT162b2 (50%) and Moderna (20%), were reported following vaccination. Most cases were reported after administration of the first (50%) and second doses (40%). Additionally, two cases were reported after receiving booster doses (fourth and fifth doses). The median interval between vaccination and onset of clinical manifestations was 2 days (IQR: 2–12), although one case presented with abrupt symptom onset within the first 5 h. The most frequent clinical manifestations were myalgia (40%), muscle weakness (40%), and dark urine (30%). A substantial proportion of patients (40%) had no prior medical history or associated comorbidities. Diagnosis was established based on clinical, laboratory, and imaging criteria. All cases showed elevated CK levels. Fifty percent of patients did not develop complications, while 25% experienced AKI. Clinical outcomes were favorable in 80% of patients. However, the remaining 20% died due to complications such as hypoxia, cardiac tamponade, multiorgan failure, massive hemorrhage, among others. Table 3 provides detailed information on the analyzed variables and study outcomes.

### 3.5. Risk of Bias and Quality

The risk of bias and methodological quality assessments were conducted according to study design and the exposure of interest, including SARS-CoV-2 infection or COVID-19 vaccination. Among the 93 case reports of rhabdomyolysis associated with SARS-CoV-2 infection, the item-level assessment using the JBI tool showed that 96.7% adequately reported patients’ demographic characteristics, 83.8% the patient history and timeline, 86.0% the clinical condition at presentation, 86.0% the diagnostic assessment and findings, 92.4% the treatment or intervention, 74.2% the post-intervention clinical condition, 74.2% adverse or unanticipated events, and 79.5% relevant lessons or learning points. Detailed assessments for each study are presented in Table S4.1.

Among the case series studies describing rhabdomyolysis associated with SARS-CoV-2 infection, the item-level assessment using the JBI tool showed that 85.7% adequately met the inclusion criterion, 57.1% the measurement criterion, 71.4% the identification criterion, 57.1% the consecutiveness criterion, 85.7% the completeness criterion, 100% the demographic reporting criterion, 14.2% the clinical information criterion, 100% the outcomes reporting criterion, 57.1% the clinical setting criterion, and 57.1% the statistical analysis criterion. Detailed item-level assessments are presented in Table S4.2.

Among the 20 case reports describing newly developed rhabdomyolysis following COVID-19 vaccination, the item-level assessment using the JBI tool showed that 100% adequately reported patients’ demographic characteristics, patient history and timeline, clinical condition at presentation, diagnostic assessment and findings, and treatment or intervention. In addition, 85.0% adequately reported the post-intervention clinical condition, whereas 100% reported adverse or unanticipated events and relevant lessons or learning points. Detailed assessments for each study are presented in Table S4.1.

Furthermore, the assessment of observational studies using the Newcastle–Ottawa Scale showed that most studies were classified as having a high risk of bias (*n* = 10; 76.9%) (Table S4.3). Overall, the case reports, case series, and observational studies provided estimates of risk of bias and methodological quality that were predominantly moderate to high. Detailed results of the risk of bias and methodological quality assessment for each included study are presented in Supplementary Material S5.

## 4. Discussion

The main findings of this systematic review indicate that rhabdomyolysis has been documented in patients with SARS-CoV-2 infection, with observational evidence suggesting that it occurs more frequently among patients with more severe disease, particularly those requiring hospitalization or intensive care. Across 13 observational studies, 818 cases were identified among 7766 patients with COVID-19, and several studies reported associations between rhabdomyolysis, acute kidney injury, and mortality; however, these findings should be interpreted as observational associations and may be influenced by disease severity, comorbidities, and other confounding factors. The case reports and case series provide complementary descriptive evidence, with a predominance of males and middle-aged adults and frequent clinical manifestations including fever, dyspnea, myalgia, and cough, together with elevated CK levels and the occurrence of AKI and other complications. These individual reports, however, are primarily hypothesis-generating and cannot establish the frequency, prognostic significance, or independent clinical contribution of these characteristics. Similarly, the 20 cases of rhabdomyolysis reported after COVID-19 vaccination, predominantly involving males and mRNA-based vaccines, describe the clinical characteristics and temporal patterns of reported events but do not permit estimation of incidence, establishment of causality, or determination of platform-specific risk. Overall, the observational evidence supports an association between rhabdomyolysis and more severe SARS-CoV-2 infection, whereas the descriptive evidence from case reports and case series provides clinical characterization and signals that warrant further investigation.

Rhabdomyolysis is a clinical and biochemical syndrome characterized by skeletal muscle injury and destruction [33]. This process results from the breakdown and necrosis of muscle tissue, leading to the release of intracellular components into the bloodstream, including CK, myoglobin, lactate dehydrogenase, and electrolytes [6,33]. Its etiology is diverse and encompasses both traumatic and non-traumatic causes, with traumatic causes being the most frequently associated with the development of rhabdomyolysis [34]. Non-traumatic causes include strenuous physical exercise, muscle hypoxia, metabolic myopathies, genetic disorders, electrolyte abnormalities, exposure to drugs and toxins, and infections [34]. Various infectious agents have been implicated in the pathogenesis of rhabdomyolysis [6]. Although infections account for approximately 5% of cases, they represent a clinically relevant etiology due to their potential to trigger severe muscle injury [35]. Among the viral agents most associated with rhabdomyolysis are influenza A virus, cytomegalovirus, herpes simplex virus, Epstein–Barr virus, Coxsackie virus, and, more recently, SARS-CoV-2 [36,37,38,39,40]. In addition, the literature has documented sporadic cases of rhabdomyolysis following vaccine administration, including vaccines against influenza and, more recently, as an adverse event associated with COVID-19 vaccines [41,42,43,44].

Rhabdomyolysis associated with SARS-CoV-2 infection may be explained by multiple interconnected pathophysiological mechanisms. The process begins with the binding of the viral spike (S) protein to ACE2 receptors expressed on host cells. Subsequently, transmembrane serine protease 2 (TMPRSS2) and the ADAM17 metallopeptidase domain participate in S protein processing, facilitating fusion between the viral and host cell membranes through the S2 subunit. As a result, SARS-CoV-2 is internalized via endocytosis, releasing its RNA into the cytoplasm for replication, translation, and subsequent assembly of new viral particles [45]. Following infection, the virus may trigger a systemic inflammatory response characterized by excessive production of pro-inflammatory cytokines, including tumor necrosis factor (TNF)-α, interleukin (IL)-1β, and IL-6, which exert deleterious effects on skeletal muscle. These cytokines disrupt muscle protein synthesis, promote anaerobic metabolism, and induce oxidative stress, thereby contributing to muscle fiber injury and, ultimately, the development of rhabdomyolysis. Furthermore, this inflammatory process may be amplified by dysregulation of the renin–angiotensin system secondary to the reduction in ACE2 expression induced by viral infection, creating a biological environment that favors muscle tissue injury and destruction [46,47]. Available evidence supports a potential role for immune-mediated muscle injury. Some studies have described inflammatory infiltrates predominantly composed of CD8+ T lymphocytes and macrophages, increased expression of major histocompatibility complex (MHC) class I, and muscle fiber degeneration in patients with severe COVID-19 [48]. In addition, binding of SARS-CoV-2 to the angiotensin-converting enzyme 2 (ACE2) receptor may contribute to a systemic inflammatory response characterized by marked elevations in cytokines, including IL-1, IL-6, and TNF-α, which may promote muscle proteolysis, mitochondrial dysfunction, vascular injury, and muscle fiber necrosis, thereby contributing to the release of intracellular components and increased CK levels [49]. Subsequent development of acute kidney injury may reflect a multifactorial process involving myoglobin-mediated tubular injury secondary to rhabdomyolysis, systemic inflammation, endothelial dysfunction, hypoxia, hypercoagulability, and macrophage activation [47]. Furthermore, a recently proposed immunological mechanism involves molecular mimicry: BLAST analysis version 2.16.0+ identified sequence similarity between the SARS-CoV-2 Spike peptide 816-SFIEDLLFNK-825 and regions of the muscle-related proteins TRIM63 (MuRF1) and CCDC63, together with the emergence of anti-TRIM63 and anti-CCDC63 autoantibodies following SARS-CoV-2 infection [50]. The proposed and described mechanisms remain hypothetical and have not yet been fully established; therefore, multicenter and multidisciplinary observational studies are needed to elucidate the underlying mechanisms.

Regarding COVID-19 vaccine–associated rhabdomyolysis, we identified 20 cases, the majority of which were linked to mRNA-based vaccines, particularly BNT162b2 (Pfizer-BioNTech) and mRNA-1273 (Moderna). Most events occurred approximately two days after vaccination, suggesting the possible involvement of immune-mediated mechanisms, whereby vaccine-induced spike proteins may share structural similarities with muscle proteins. Nevertheless, these events were exceptionally rare compared with the billions of vaccine doses administered worldwide, supporting the strong safety profile of COVID-19 vaccines. From a pathophysiological perspective, mRNA-based vaccines delivered through lipid nanoparticles (LNPs) induce a localized innate immune response at the injection site, characterized by activation of type I interferon (IFN-I)-dependent pathways and maturation of antigen-presenting cells. In susceptible individuals, this response may promote the development of idiosyncratic myopathic events; however, the available evidence does not support the existence of a generalized myotoxic effect associated with these vaccine platforms [14,51,52,53]. Recent data show that mRNA and Spike protein derived from the BNT162b2 and mRNA-1273 vaccines can be detected locally in the deltoid muscle shortly after vaccination, with fibroblasts and muscle satellite cells identified as important sources of Spike protein expression, supporting the possibility that the injected tissue may directly participate in antigen expression and local inflammatory signaling [54]. Molecular mimicry remains a biologically plausible hypothesis, particularly because epitope-mapping studies have identified sequence similarities between the SARS-CoV-2 Spike fusion peptide (816-SFIEDLLFNK-825) and epitopes of TRIM63 and CCDC63, together with increased levels of anti-TRIM63 and anti-CCDC63 autoantibodies following SARS-CoV-2 infection; however, these findings were demonstrated in the context of infection and do not establish that the same mechanism causes vaccine-associated rhabdomyolysis [50]. Therefore, molecular mimicry should be considered one of several testable hypotheses rather than an established mechanism.

In multinational safety assessments, reproducible associations related to COVID-19 vaccination have been primarily concentrated in conditions such as myocarditis, pericarditis, and Guillain–Barré syndrome, with no consistent evidence supporting a reproducible association with muscle injury. Consequently, the benefit–risk profile of COVID-19 vaccination remains clearly favorable. Consistent with these findings, the Vaccine Adverse Event Reporting System (VAERS) recorded 591 cases of rhabdomyolysis following COVID-19 vaccination. However, considering the billions of vaccine doses administered worldwide, these events remain exceptionally rare. Moreover, the benefits of vaccination in preventing severe forms of COVID-19 substantially outweigh the potential risks, while cases of vaccine-associated rhabdomyolysis are generally uncommon, transient, and potentially treatable. Taken together, these findings support the continued recommendation of COVID-19 vaccination while underscoring the importance of maintaining robust pharmacovigilance systems and evidence-based communication strategies to address public concerns and strengthen confidence in immunization programs [55,56,57,58].

From a public health perspective, our findings may have implications for epidemiological surveillance and clinical practice. The documentation of rhabdomyolysis among patients with SARS-CoV-2 infection highlights the importance of clinical recognition and appropriate evaluation, particularly in hospitalized patients with severe disease or compatible symptoms. Although CK assessment may be clinically considered in patients with symptoms suggestive of muscle injury, severe disease, renal dysfunction, or other relevant risk factors, this review did not directly evaluate screening strategies or determine whether routine CK monitoring improves clinical outcomes. Therefore, this should be regarded as a potential clinical implication rather than an evidence-based recommendation derived directly from the studies included in this review. Future research should focus on population-based comparative studies incorporating standardized CK definitions and stratification according to relevant comorbidities and exposures, such as statin use. This approach will allow for more precise estimation of absolute risk and identification of potentially susceptible subgroups, while avoiding overestimation of an event that, according to the available evidence, remains clearly uncommon [55,56,59,60,61].

The methodological quality of the included studies should also be considered when interpreting the findings of this review. Although the assessments using the JBI and Newcastle–Ottawa tools showed variable adherence to methodological criteria, a substantial proportion of the evidence was derived from low-quality observational studies and descriptive reports, particularly case reports and case series. These characteristics limit the ability to establish causal associations, adequately control for confounding factors, and obtain precise estimates of the frequency of rhabdomyolysis. Accordingly, quantitative estimates, when available, should be interpreted with caution and in conjunction with the clinical and methodological heterogeneity across studies; similarly, descriptive findings should be regarded primarily as evidence that rhabdomyolysis has been documented in association with SARS-CoV-2 infection or following vaccination, rather than as sufficient evidence to establish the magnitude of risk, prognosis, or causality. In this context, the methodological quality and study designs reduce the certainty of the conclusions and support a cautious interpretation of the findings.

### 4.1. Strengths and Limitations

Our review has several methodological strengths that enhance the robustness and reliability of our findings. We conducted a comprehensive search of PubMed, Scopus, EMBASE, and Web of Science to identify relevant studies and adhered to the PRISMA 2020 guidelines to ensure transparent and systematic reporting. We included 133 studies, representing one of the largest bodies of evidence on SARS-CoV-2-related rhabdomyolysis to date. Study quality was assessed using the JBI and Newcastle-Ottawa Scale tools, and a random-effects meta-analytical model was employed to account for between-study heterogeneity.

This study provides a comprehensive and up-to-date synthesis of the available evidence on the global incidence and prevalence of rhabdomyolysis associated with SARS-CoV-2 infection. Additionally, we included studies originally designed for other purposes that reported extrapulmonary manifestations or complications associated with SARS-CoV-2 infection. However, our study has several limitations. First, this systematic review included case reports and case series because of the limited availability of original studies investigating rhabdomyolysis following COVID-19 vaccination. These study designs are susceptible to selection, reporting, and publication biases and do not allow causal inferences or reliable estimation of incidence or prevalence; therefore, their findings should be interpreted with caution. Second, the eligibility criteria were restricted to studies published in English, which may have resulted in the exclusion of potentially relevant studies published in other languages. Third, rhabdomyolysis may have been underreported across countries and healthcare settings. In addition, diagnostic bias may have affected the identification of cases, particularly for vaccine-associated events, as increased awareness of a potential adverse event may result in preferential recognition and reporting. Fourth, the included studies showed substantial clinical and methodological heterogeneity, including differences in patient populations, disease severity, hospitalization status, clinical settings, follow-up, and criteria used to identify or diagnose rhabdomyolysis. These differences likely contributed to the variability observed across studies. Because of the limited availability of adequately stratified data, additional subgroup analyses could not be performed to determine the relative contribution of these potential sources of heterogeneity. Although quantitative synthesis was initially considered, the statistical analysis was removed because the observed heterogeneity was extreme (I^2^ = 100%) and the resulting pooled proportions were considered potentially unreliable and susceptible to overestimation. Accordingly, the reported proportions should be interpreted descriptively rather than as precise estimates of the frequency or risk of rhabdomyolysis associated with SARS-CoV-2 infection or COVID-19 vaccination. Finally, the relatively small number of patients and studies evaluating rhabdomyolysis following COVID-19 vaccination limits the generalizability of these findings. This may partly reflect the emerging nature of this potential adverse event; however, the available evidence remains insufficient to estimate the incidence or platform-specific risk. Although the findings support an association between SARS-CoV-2 infection and the occurrence of rhabdomyolysis, the underlying causal mechanisms remain incompletely understood. Likewise, reports of rhabdomyolysis following COVID-19 vaccination do not establish causality. Therefore, the findings should be interpreted within the methodological limitations of the available evidence and without extrapolating beyond the populations and clinical settings represented in the included studies.

### 4.2. Recommendations and Future Research Agenda

Large-scale, multicenter, and multidisciplinary observational studies are needed to further elucidate the underlying mechanisms of rhabdomyolysis associated with SARS-CoV-2 infection and COVID-19 vaccination. Detailed analyses of cytokines, inflammatory mediators, and other molecular biomarkers could contribute to a more comprehensive characterization of the disease and improve understanding of its pathophysiology. Future research should also focus on prospective studies designed to accurately estimate the incidence of rhabdomyolysis across different levels of COVID-19 severity and diverse demographic populations. In addition, investigations incorporating muscle biopsies and advanced molecular profiling may help clarify the biological pathways linking SARS-CoV-2 infection to skeletal muscle injury.

Comparative studies evaluating different therapeutic strategies for COVID-19-related rhabdomyolysis are warranted to optimize clinical management and improve patient outcomes. For vaccine-associated cases, well-designed case–control studies with appropriate temporal controls are necessary to establish causal relationships and identify potential risk factors. Furthermore, long-term follow-up studies assessing persistent muscle dysfunction among COVID-19 survivors could facilitate the development of targeted rehabilitation strategies and improve understanding of the broader musculoskeletal consequences of the pandemic. Finally, the development of standardized diagnostic criteria, reporting frameworks, and evidence-based clinical practice guidelines is essential to improve the diagnosis, management, and surveillance of rhabdomyolysis associated with SARS-CoV-2 infection and COVID-19 vaccination, thereby supporting more effective clinical decision-making and strengthening public health responses to emerging infectious diseases.

## 5. Conclusions

This review shows that rhabdomyolysis has been documented in patients with SARS-CoV-2 infection. However, the reported proportions vary widely across highly heterogeneous cohorts, which predominantly included hospitalized patients, often with severe disease or admission to intensive care units, particularly during the early phases of the pandemic. Therefore, these findings should not be generalized to all individuals with SARS-CoV-2 infection, contemporary variants, vaccinated populations, or patients managed in the community, and the true frequency of rhabdomyolysis remains uncertain. Rhabdomyolysis may be considered in the differential diagnosis of patients with acute muscle injury during or after SARS-CoV-2 infection. Cases of rhabdomyolysis following COVID-19 vaccination have also been reported; however, case reports and case series do not allow a causal relationship to be established or the incidence or platform-specific risk to be determined. Continued pharmacovigilance and clinical awareness are needed to facilitate the timely recognition and appropriate management of suspected cases.

## Figures and Tables

**Table 1:** New-onset rhabdomyolysis associated with SARS-CoV-2 infection (*n* = 133 patients).

Variable	Results	Variable	Results
Age Median (IQR)	45.5 (24–62) years	Alterations of laboratory parameters and *n* (%)	CK: 129 (96.9) AST: 81 (60.9) Creatinine: 52 (39.1) ALT: 12 (9.0)
Sex *n* (%)	Male: 97 (72.9) Female: 35 (26.3) NR: 1 (0.8)	Complication *n* (%)	AKI/Renal failure: 22 (16.5) Cardiac events: 10 (7.5) Neurologic events: 4 (3.0) Myositis: 4 (3.0) Multiorgan failure: 4 (3.0)
Clinical Manifestations *n* (%)	Fever: 72 (54.1) Dyspnea: 55 (41.4) Myalgia: 51 (38.3) Cough: 49 (36.8) Weakness: 32 (24.1) Dark urine: 20 (15.0) Fatigue/Asthenia: 16 (12.0) Limb pain: 14 (10.5)	Treatment *n* (%)	IV fluids/fluid therapy: 60 (45.1) Steroids: 34 (25.6) Antibiotics: 30 (22.6) Hydroxychloroquine: 19 (14.3) Renal replacement therapies: 19 (14.3)
Background- comorbidities *n* (%)	HBP: 43 (32.3) DM: 23 (17.3) Obesity: 15 (11.3) CKD: 14 (10.5) Dyslipidemia: 10 (7.5) None: 36 (27.1)	Development *n* (%)	Favorable: 96 (72.2) Death: 31 (23.3) Unfavorable: 2 (1.5) NR: 4 (3.0)

AKI: acute kidney injury; ALT: alanine aminotransferase; AST: aspartate aminotransferase; CK: creatine kinase; CKD: chronic kidney disease; DM: diabetes mellitus; HBP: high blood pressure; IHD: ischemic heart disease; IQR: interquartile range; IV: intravenous; NR: not reported.

**Table 2:** Characteristics of observational studies on Rhabdomyolysis and SARS-CoV-2 infection.

First Author/Year	Country	Design	Period	Age Group	Age	Total Sample	Cases *	Sex Among Cases (M/F)	CK (IU/L)	AKI *n* (%) ***	Main Results
Albaba et al. 2021 [20]	USA	Cohort	March 2020–January 2021	All ages	62 (47–75) **	240	22	18/4	3099	13 (59.1)	Rhabdomyolysis was 9.2% in hospitalized patients with COVID-19.
Ali et al. 2021 [21]	Qatar	Cohort	January–December 2020	≥18 years	52 (22–86) **	413	65	NR	>1000	41 (63.07)	Rhabdomyolyses are important complications among COVID-19 hospitalized patients
Geng et al. 2021 [22]	China	Cohort	February–April 2020	All ages	58.7 ± 14.85	1014	22	NR	>1000	17 (77.27)	2.2% of patients developed rhabdomyolysis following SARS-CoV-2 infection
Haroun et al. 2021 [23]	USA	Cohort	March–May 2020	≥18 years	68 (21–93) **	825	140	90/50	1323 (775–2848) **	112 (80)	Rhabdomyolysis was a common finding among hospitalized patients with COVID-19
Martinot et al. 2021 [24]	France	Cohort	March 2020	All ages	NR	600	5	NR	>1000	NR	Extrapulmonary complications occurred in ICU patients
Mohamed et al. 2021 [25]	USA	Cohort	March 2020	All ages	NR	575	7	NR	>2000	7 (100)	Rhabdomyolysis is a cause of AKI in patients with COVID-19
Mokhtari et al. 2021 [26]	USA	Cohort	March–April 2020	≥18 years	57 (45–67) **	235	114	84/30	2672 (1607–4755) **	94 (82.5)	COVID-19 patients nearly half developed rhabdomyolysis
Zamoner et al. 2021 [27]	Brazil	Cohort	March 2020	≥18 years	61.62 ± 14.52	101	10	NR	NR	NR	10.2% of patients developed rhabdomyolysis following SARS-CoV-2 infection
Khatib et al. 2022 [28]	Qatar	Cohort	March–July 2020	≥18 years	50 (41–59) **	1079	189	NR	NR	NR	Rhabdomyolysis as a possible etiology of mortality in patients with COVID-19
Nashwan et al. 2023 [29]	Qatar	Cohort	March–July 2020	≥18 years	51 ± 15.0	1079	146	140/6	2663.87 ± 3308.13	59 (40.4)	Rhabdomyolysis increases the risk of death in COVID-19 patients
Hashemi et al. 2024 [30]	Iran	Cross-sectional	March 2020–March 2021	All ages	56.11 ± 18.36	506	44	33/11	2338.32 ± 1509.71	NR	8.69% of patients developed rhabdomyolysis following SARS-CoV-2 infection
Murt and Altiparmak 2024 [31]	Türkiye	Cohort	March–June 2020	NR	61.00 ± 19.10	115	15	11/4	1929.30 ± 1282.20	15	Rhabdomyolysis was present in 13% of patients with COVID-19.
Samardzic et al. 2024 [32]	USA	Cohort	March 2020–May 2021	≥18 years	61 ± 17.9	984	39	NR	NR	27 (69.2)	4% of patients developed rhabdomyolysis following SARS-CoV-2 infection

* Number of cases of rhabdomyolysis after SARS-CoV-2 infection. ** Range, between minimum and maximum value. *** AKI percentages were calculated using the number of rhabdomyolysis cases as the denominator. AKI: acute kidney injury; CK: creatine kinase; F: female; ICU: intensive care unit; M: male; NR: not reported; SD: standard deviation.

**Table 3:** New-onset rhabdomyolysis associated with COVID-19 vaccination (*n* = 20 patients).

Variable	Results	Variable	Results
Age Median (IQR)	52.5 (22.5–76.5) years	Time to symptom onset Median (IQR)	2 (2–12) days
Sex *n* (%)	Male: 15 (75) Female: 5 (25)	Clinical Manifestations *n* (%)	Myalgia/Muscle pain: 8 (40) Weakness: 8 (40) Dark urine: 6 (30) Swelling / Edema: 3 (15) SOB / Dyspnea: 3 (15) GI symptoms: 3 (15)
Type of vaccine *n* (%)	Pfizer BioNTech 162b2: 10 (50) Moderna: 4 (20) Oxford/AstraZeneca: 4 (20) Johnson & Johnson: 1 (5) NR: 1 (5)	Background-comorbidities *n* (%)	None: 8 (40) Asthma: 4 (20) HBP: 4 (20) DM: 4 (20) Dyslipidemia: 4 (20) IHD: 3 (15) CVD/CVA: 2 (10)
Vaccine technologies *n* (%)	mRNA: 15 (75) viral vector: 5 (25)	Alterations of laboratory parameters and *n* (%)	CK: 20 (100) AST: 8 (40) ALT: 5 (25) Creatinine: 5 (25)
Dose *n* (%)	1st: 10 (50) 2nd: 8 (40) 4th: 1 (5) 5th: 1 (5)	Complication *n* (%)	None (without complications): 10 (50) AKI: 5 (25) ANCA-vasculitis/Glomerulonephritis: 2 (10)
Development *n* (%)	Favorable: 16 (80) Death: 4 (20)	Treatment *n* (%)	Intravenous hydration: 12 (60) Corticosteroids: 9 (45) IVIG: 4 (20) Acetaminophen: 3 (15)

AKI: acute kidney injury; ALT: alanine aminotransferase; ANCA: antineutrophil cytoplasmic antibody; AST: aspartate aminotransferase; CK: creatine kinase; CVA: cerebrovascular accident; CVD: cerebrovascular disease; DM: diabetes mellitus; GI: gastrointestinal; HBP: high blood pressure; IHD: ischemic heart disease; IQR: interquartile range; IVIG: intravenous immunoglobulin; NR: not reported; SOB: shortness of breath.

## Data Availability

No new data were created or analyzed in this study. Data sharing is not applicable to this article.

## Supplementary Materials

The following supporting information can be downloaded at: https://www.mdpi.com/article/10.3390/medicina62091769/s1, Supplementary Material S1: Search Strategy; Table S1: New-onset rhabdomyolysis induced by SARS-CoV-2 infection; Table S2: Definition and ascertainment of rhabdomyolysis among included observational studies; Table S3: New-onset Rhabdomyolysis induced by COVID-19 vaccines; Supplementary Material S5 (Table S4.1: JBI tool for assessing risk of bias and quality of case report studies, Table S4.2: JBI tool for assessing risk of bias and quality of case series studies, Table S4.3:Evaluation of observational studies using NOS).

## Abbreviations

The following abbreviations are used in this manuscript:

ACE2	Angiotensin-Converting Enzyme 2
AKI	Acute Kidney Injury
ALT	Alanine Aminotransferase
AST	Aspartate Aminotransferase
CI	Confidence Intervals
CK	Creatine Kinase
COVID-19	Coronavirus Disease 2019
ICU	Intensive Care Unit
IQR	Interquartile Range
NOS	Newcastle–Ottawa Scale
SARS-CoV-2	Severe Acute Respiratory Syndrome Coronavirus 2
VAERS	Vaccine Adverse Event Reporting System
WHO	World Health Organization
