# Supplementary material for: Rhabdomyolysis Reported in Association with SARS-CoV-2 Infection and COVID-19 Vaccination: A Systematic Review
Source: Medicina (Kaunas). 2026 Sep 15;62(9):1769. doi: 10.3390/medicina62091769 (PMC13608938; doi:10.3390/medicina62091769)
Supplement: Supplementary file 1 [file medicina-62-01769-s001.zip › SM 1. Search Strategy.pdf]

Supplementary material S1.

Search Strategy

Databases: PubMed, Scopus, EMBASE and Web of Science platform.

December 24, 2025

PubMed (n=249)

#1: ("COVID-19"[TIAB] OR "COVID 19" [TIAB] OR "2019-nCoV Infection"[TIAB] OR "2019 nCoV Infection"[TIAB] OR "2019-nCoV Infections"[TIAB] OR "SARS-CoV-2 Infection"[TIAB] OR "Infection, SARS-CoV-2"[TIAB] OR "SARS CoV 2 Infection"[TIAB] OR "SARS-CoV-2 Infections"[TIAB] OR "2019 Novel Coronavirus Disease"[TIAB] OR "2019 Novel Coronavirus Infection"[TIAB] OR "2019-nCoV Disease"[TIAB] OR "2019 nCoV Disease"[TIAB] OR "2019-nCoV Diseases"[TIAB] OR COVID19[TIAB] OR "Coronavirus Disease 2019"[TIAB] OR "Coronavirus Disease-19"[TIAB] OR "Coronavirus Disease 19"[TIAB] OR "Severe Acute Respiratory Syndrome Coronavirus 2 Infection"[TIAB] OR "COVID-19 Virus Disease"[TIAB] OR "COVID 19 Virus Disease"[TIAB] OR "SARS Coronavirus 2 Infection"[TIAB] OR "COVID-19 Virus Infection"[TIAB] OR "COVID 19 Virus Infection"[TIAB] OR "COVID-19 Virus Infections"[TIAB] OR "COVID-19 Pandemic"[TIAB] OR "COVID 19 Pandemic"[TIAB] OR "COVID-19 Pandemics"[TIAB]):
416,391 results

#2: ("COVID-19 Vaccines"[TIAB] OR "COVID 19 Vaccines"[TIAB] OR "SARS2 Vaccines"[TIAB] OR "SARS2 Vaccine"[TIAB] OR "Coronavirus Disease 2019 Vaccine"[TIAB] OR "Coronavirus Disease-19 Vaccine"[TIAB] OR "Coronavirus Disease 19 Vaccine"[TIAB] OR "COVID 19 Vaccine"[TIAB] OR "2019-nCoV Vaccine"[TIAB] OR "2019 nCoV Vaccine"[TIAB] OR "SARS-CoV-2 Vaccine"[TIAB] OR "SARS CoV 2 Vaccine"[TIAB] OR "2019-nCoV Vaccines"[TIAB] OR "2019 nCoV Vaccines"[TIAB] OR "COVID-19 Vaccine"[TIAB] OR "SARS Coronavirus 2 Vaccines"[TIAB] OR "COVID19 Vaccines"[TIAB] OR "COVID19 Vaccine"[TIAB] OR "SARS-CoV-2 Vaccines"[TIAB] OR "SARS CoV 2 Vaccines"[TIAB] OR "2019 Novel Coronavirus Vaccine"[TIAB]): 26,694 results

#3: (Rhabdomyolysis[TIAB] OR Rhabdomyolysis[MeSH] OR Rhabdomyolysis[OT]): 12,150 results

#4: (#1 OR #2) AND #3: 249 results

Scopus (n=538)

#1: TITLE-ABS-KEY(("COVID-19" OR "COVID 19" OR "2019-nCoV Infection" OR "2019 nCoV Infection" OR "2019-nCoV Infections" OR "SARS-CoV-2 Infection" OR "Infection, SARS-CoV-2" OR "SARS CoV 2 Infection" OR "SARS-CoV-2 Infections" OR "2019 Novel Coronavirus Disease" OR "2019 Novel Coronavirus Infection" OR "2019-nCoV Disease" OR "2019 nCoV Disease" OR "2019-nCoV Diseases" OR "COVID19" OR "Coronavirus Disease 2019" OR "Coronavirus Disease-19" OR "Coronavirus Disease 19" OR "Severe Acute Respiratory Syndrome Coronavirus 2 Infection" OR "COVID-19 Virus Disease" OR "COVID 19 Virus Disease" OR "SARS Coronavirus 2 Infection" OR "COVID-19 Virus Infection" OR "COVID 19 Virus Infection" OR "COVID-19 Virus Infections" OR "COVID-19 Pandemic" OR "COVID 19 Pandemic" OR "COVID-19 Pandemics")): 713,862 results

#2: TITLE-ABS-KEY(("COVID-19 Vaccines" OR "COVID 19 Vaccines" OR "SARS2 Vaccines" OR "SARS2 Vaccine" OR "Coronavirus Disease 2019 Vaccine" OR "Coronavirus Disease-19 Vaccine" OR "Coronavirus Disease 19 Vaccine" OR "COVID 19 Vaccine" OR "2019-nCoV Vaccine" OR "2019 nCoV Vaccine" OR "SARS-CoV-2 Vaccine" OR "SARS CoV 2 Vaccine" OR "2019-nCoV Vaccines" OR "2019 nCoV Vaccines" OR "COVID-19 Vaccine" OR "SARS Coronavirus 2 Vaccines" OR "COVID19 Vaccines" OR "COVID19 Vaccine" OR "SARS-CoV-2 Vaccines" OR "SARS CoV 2 Vaccines" OR "2019 Novel Coronavirus Vaccine")): 53, 434 results

#3: TITLE-ABS-KEY(("Rhabdomyolysis")): 20,801 results

#4: (#1 OR #2) AND #3: 538 results

EMBASE (n=684)

#1: ('covid-19'/exp OR 'covid-19' OR 'covid 19'/exp OR 'covid 19' OR '2019-ncov infection'/exp OR '2019-ncov infection' OR '2019 ncov infection'/exp OR '2019 ncov infection' OR '2019-ncov infections' OR 'sars-cov-2 infection'/exp OR 'sars-cov-2 infection' OR 'infection, sars-cov-2' OR 'sars cov 2 infection'/exp OR 'sars cov 2 infection' OR 'sars-cov-2 infections' OR '2019 novel coronavirus disease'/exp OR '2019 novel coronavirus disease' OR '2019 novel coronavirus infection'/exp OR '2019 novel coronavirus infection' OR '2019-ncov disease'/exp OR '2019-ncov disease' OR '2019 ncov disease'/exp OR '2019 ncov disease' OR '2019-ncov diseases' OR 'covid19'/exp OR 'covid19' OR 'coronavirus disease 2019'/exp OR 'coronavirus disease 2019' OR 'coronavirus disease-19'/exp OR 'coronavirus disease-19' OR 'coronavirus disease 19'/exp OR 'coronavirus disease 19' OR 'severe acute respiratory syndrome coronavirus 2 infection'/exp OR 'severe acute respiratory syndrome coronavirus 2 infection' OR 'covid-19 virus disease' OR 'covid 19 virus disease' OR 'sars coronavirus 2 infection'/exp OR 'sars coronavirus 2 infection' OR 'covid-19 virus infection' OR 'covid 19 virus infection' OR 'covid-19 virus infections' OR 'covid-19 pandemic' OR 'covid 19 pandemic' OR 'covid-19 pandemics'): 533,548 results

#2: ('covid-19 vaccines' OR 'covid 19 vaccines' OR 'sars2 vaccines' OR 'sars2 vaccine' OR 'coronavirus disease 2019 vaccine' OR 'coronavirus disease-19 vaccine' OR 'coronavirus disease 19 vaccine' OR 'covid 19 vaccine' OR '2019-ncov vaccine' OR '2019 ncov vaccine' OR 'sars-cov-2 vaccine' OR 'sars cov 2 vaccine' OR '2019-ncov vaccines' OR '2019 ncov vaccines' OR 'covid-19 vaccine' OR 'sars coronavirus 2 vaccines' OR 'covid19 vaccines' OR 'covid19 vaccine' OR 'sars-cov-2 vaccines' OR 'sars cov 2 vaccines' OR '2019 novel coronavirus vaccine'): 49,893 results

#3: ('rhabdomyolysis'): 23,880 results

#4: (#1 OR #2) AND #3: 679 results

Web of Science (WOS) (n= 196)

#1: (TS=("COVID-19" OR "COVID 19" OR "2019-nCoV Infection" OR "2019 nCoV Infection" OR "2019-nCoV Infections" OR "SARS-CoV-2 Infection" OR "Infection, SARS-CoV-2" OR "SARS CoV 2 Infection" OR "SARS-CoV-2 Infections" OR "2019 Novel Coronavirus Disease" OR "2019 Novel Coronavirus Infection" OR "2019-nCoV Disease" OR "2019 nCoV Disease" OR "2019-nCoV Diseases" OR "COVID19" OR "Coronavirus Disease 2019" OR "Coronavirus Disease-19" OR "Coronavirus Disease 19" OR "Severe Acute Respiratory Syndrome Coronavirus 2 Infection" OR "COVID-19 Virus Disease" OR "COVID 19 Virus Disease" OR "SARS Coronavirus 2 Infection" OR "COVID-19 Virus Infection" OR "COVID 19 Virus Infection" OR "COVID-19 Virus Infections" OR "COVID-19 Pandemic" OR "COVID 19 Pandemic" OR "COVID-19 Pandemics")): 559,742 results

#2: (TS=("COVID-19 Vaccines" OR "COVID 19 Vaccines" OR "SARS2 Vaccines" OR "SARS2 Vaccine" OR "Coronavirus Disease 2019 Vaccine" OR "Coronavirus Disease-19 Vaccine" OR "Coronavirus Disease 19 Vaccine" OR "COVID 19 Vaccine" OR "2019-nCoV Vaccine" OR "2019 nCoV Vaccine" OR "SARS-CoV-2 Vaccine" OR "SARS CoV 2 Vaccine" OR "2019-nCoV Vaccines" OR "2019 nCoV Vaccines" OR "COVID-19 Vaccine" OR "SARS Coronavirus 2 Vaccines" OR "COVID19 Vaccines" OR "COVID19 Vaccine" OR "SARS-CoV-2 Vaccines" OR "SARS CoV 2 Vaccines" OR "2019 Novel Coronavirus Vaccine")): 29,050 results

#3: (KP=("Rhabdomyolysis") OR AK=("Rhabdomyolysis") OR TI=("Rhabdomyolysis")): 6,921 results

#4: (#1 OR #2) AND #3: 196 results
