# Supplementary material for: Rhabdomyolysis Reported in Association with SARS-CoV-2 Infection and COVID-19 Vaccination: A Systematic Review
Source: Medicina (Kaunas). 2026 Sep 15;62(9):1769. doi: 10.3390/medicina62091769 (PMC13608938; doi:10.3390/medicina62091769)
Supplement: Supplementary file 1 [file medicina-62-01769-s001.zip › SM 2. Table S1.pdf]

Table S1. New-onset rhabdomyolysis induced by SARS-CoV-2 infection.

First author name/year of publication	Country	Design	Age/Sex	Background-comorbidities	Time to symptom onset	Clinical Manifestations	Lab tests	Imaging	Diagnostic	Complication	Treatment	Development
Alrubaye and Choudhary 2020[1]	USA	Case report	35 F	NR	NR	Fever<br>Myalgia<br>Cough	CK: 29000 U/L<br>CK-MB: 499.7 µg/mL<br>AST: 475 U/L	Normal	Rhabdomyolysis	None	Ringer's lactate<br>Normal saline	Favorable
Anklesaria et al. 2020[2]	USA	Case report	57 M	Obesity<br>HBP<br>Dyslipidemia	NR	Malaise<br>Fever<br>Generalized weakness<br>Myalgia	CK: 923120 U/L<br>AST: 3995 U/L<br>CRP: > 32 mg/dL	NR	Rhabdomyolysis	Ventricular arrythmias	Calcium chloride<br>ACLS	Death
Aria et al. 2020[3]	Iran	Case report	50 M	Hypothyroidism	NR	Headache<br>Vomiting<br>Nausea<br>Epigastric pain<br>Loss of consciousness	CK: 41240 U/L<br>AST: 850 U/L	MRI: micro-hemorrhaging in basal ganglia and downward cerebellar tonsile herniation	Rhabdomyolysis	Brain death	Atropine<br>Chloroquine<br>Lopinavir/Ritonavir<br>Vancomycin	Death

Anwar and Al Lawati 2020[4]	Oman	Case report	16 M	None	NR	Myalgia<br>Fever<br>Sore throat<br>Leg weakness<br>Inability to walk	NR	NR	Rhabdomyolysis	Myositis	NR	Death
Uysal et al. 2020[5]	Turkey	Case report	60 M	None	NR	Myalgia<br>Fatigue	CK: 4267 U/L<br>AST: 137 U/L<br>LDH: 781 U/L	CT: small ground-glass nodules were seen	Rhabdomyolysis	None	Hydroxychloroquine<br>Oseltamivir<br>Azithromycin<br>Favipiravir<br>Antiviral	Favorable
Buckholz et al. 2020[6]	USA	Case series	43 M	None	NR	Myalgia<br>Cough<br>Fever	CK: 75240 U/L<br>Creatinine: 12.3 mg/dL<br>AST: 1274 U/L	CXR: bilateral pneumonia	Rhabdomyolysis	NR	Hydroxychloroquine<br>Tocilizumab<br>Methylprednisolone	Favorable
Buckholz et al. 2020[6]	USA	Case series	37 M	None	NR	Fever<br>Dyspnea<br>Myalgia	CK: 82960 U/L<br>AST: 902 U/L	CXR: bilateral hazy opacities	Rhabdomyolysis	NR	Dexamethasone	Favorable
Buckholz et al. 2020[6]	USA	Case series	75 M	Deep vein thrombosis	NR	Rhinorrhea<br>Back pain<br>Weakness	CK: 3638 U/L<br>Creatine: 1.78 mg/dL	None	Rhabdomyolysis	NR	None	Favorable

Buckholz et al. 2020[6]	USA	Case series	59 M	None	NR	Cough Fever Diarrhea	CK: 8310 U/L AST: 186 U/L	CXR: patchy bilateral pneumonia	Rhabdomyolysis	NR	Hydroxychloroquine Remdesivir	Death
Buckholz et al. 2020[6]	USA	Case series	66 M	HBP	NR	Cough Fever SOB	CK: 10100U/L AST: 263 U/L	CXR: bilateral multifocal pneumonia	Rhabdomyolysis	NR	Hydroxychloroquine	Favorable
Buckholz et al. 2020[6]	USA	Case series	70 F	CKD Multiple myeloma	NR	SOB Cough Malaise	CK: 406300 U/L AST: >6000 U/L Creatinine: 12.3 mg/dL	CXR: bilateral multifocal pneumonia	Rhabdomyolysis	NR	Hydroxychloroquine	Death
Case et al. 2020[7]	USA	Case report	63 M	HBP	NR	Fever Chills Cough Epigastric pain	CK: 20000 U/L Creatinine: 2.3 mg/dL	CT: scant peripheral ground-glass infiltrates Echo: pericardial effusion	Rhabdomyolysis	Systemic capillary leak syndrome	Crystalloid resuscitation Antibiotics Vasopressors Intubation Mechanical ventilation	Death
Chan et al. 2020[8]	USA	Case report	75 F	CAD HBP	NR	Weakness Decrease in appetite	CK: 2767 U/L	CXR: left lower lobe infiltrates	Rhabdomyolysis	NR	IV fluids	Favorable
Chan et al. 2020[8]	USA	Case report	71 M	HBP CKD	NR	Twitching Weakness	CK: 1859 U/L Creatinine: 5.6 mg/dL	CXR: signs of multifocal pneumonia	Rhabdomyolysis	NR	Doxycycline Ceftriaxone Hydroxychloroquine	NR

Chedid et al. 2020[9]	USA	Case report	51 M	HBP<br>DM2<br>CKD<br>Obstructive sleep apnea	NR	Myalgias<br>Cough<br>Chills	CK: 464000 U/L<br>Creatinine: 19.1mg/dL<br>AST: 1173 U/L	CXR: ill-defined left basilar opacities read	Rhabdomyolysis	Kidney failure	IV fluids<br>Chlorothiazide<br>Furosemide	Favorable
Chong and Saha 2020[10]	USA	Case report	37 M	None	NR	Dyspnea<br>Fatigue	CK: 35000 U/L<br>Creatinine: 5 mg/dL	NR	Rhabdomyolysis	NR	IV fluids<br>RRT	Death
Cunha et al. 2020[11]	Portugal	Case report	46 F	Breast cancer	16 days	Vomiting<br>Abdominal pain<br>Fever	CK: 87456 U/L<br>AST: 442 U/L	CT-scan: diffuse bilateral ground-glass opacities	Rhabdomyolysis	NR	Paracetamol<br>Methylprednisolone<br>Hydroxychloroquine<br>IV bicarbonate	Favorable
Foster et al. 2020[12]	USA	Case report	40 M	HIV<br>HBP	2 weeks	Fever<br>Chills<br>Cough productive of grayish sputum<br>Fatigue<br>Leg cramping<br>Poor appetite.	Creatinine: 17.4 mg /dL<br>CK: 2185 U/L	CXR: patchy peripheral infiltrates	Rhabdomyolysis	AKI	Hemodialysis	Favorable
Fujimaru et al. 2020[13]	Japan	Case report	45 M	Smoker	NR	Respiratory failure<br>Dyspnea	CK: 79508 U/L	CT: ground-glass opacities with	Rhabdomyolysis	AKI	Mechanical ventilation	Favorable

						Brown colored urine	Creatinine: 8.1 mg/dL	bilateral peripheral distribution			Piperacillin-tazobactam Azithromycin Favipiravir	
Gefen et al. 2020[14]	USA	Case report	16	M	Morbid obesity Eczema	NR	Fever Myalgias SOB with exertion Dark urine	CK: 427.656 U/L AST: 839 U/L	Normal	Rhabdomyolysis	NR	IV fluids Acetaminophen Morphine	Favorable
Husain et al. 2020[15]	USA	Case report	38	M	NR	NR	Fever Cough SOB Myalgia	CK: 33000 U/L Creatinine: 1.5 mg/dL	CT: bilateral scattered ground glass opacities, empyema	Rhabdomyolysis	Multi organ failure	Heparin	Favorable
Jin and Tong. 2020[16]	China	Case report	60	M	NR	NR	Cough Fever Pain and weakness in the lower extremities	CK: 17434 U/L AST: 373 U/L	CT: both lungs were covered with ground-glass shadows	Rhabdomyolysis	None	NR	NR
Kajani et al. 2020[17]	USA	Case report	-	M	Schizophrenia Hepatitis C	NR	Altered mental status Fever Tachycardia Hypoxia Tachypnea	CK: 120000 U/L Creatinine: 5.8 mg/dL	CXR: bilateral peripheral infiltrates	Rhabdomyolysis	Neuroleptic malignant syndrome	Antibiotics Antiviral Hydroxychloroquine Intubation Dantrolene	NR
Khosla et al. 2020[18]	USA	Case series	65	M	HBP Hyperlipidemia	NR	SOB Cough Diarrhea	CK: 7854 U/L Creatinine: 5.3 mg/dL	NR	Rhabdomyolysis	NR	Anticoagulation Azithromycin Tocilizumab	Death
Khosla et al. 2020[18]	USA	Case series	78	M	DM2 HBP	NR	Severe mitral valve regurgitation	CK: > 22000 U/L	NR	Rhabdomyolysis	Sepsis/septic shock	Pressor support Methylprednisolone	Death

				Hyperlipidemia		Heart failure					Hydroxychloroquine	
Khosla et al. 2020[18]	USA	Case series	67 M	HBP<br>Hyperlipidemia<br>DM2	NR	Altered mentation<br>Hypotension<br>Hypoxia	Creatinine: 24.3 mg/dL<br>CK: 6164U/L	NR	Rhabdomyolysis	None	Hydroxychloroquine<br>Azithromycin<br>Steroids	Favorable
Khosla et al. 2020[18]	USA	Case series	58 M	DM2<br>HBP<br>Hyperlipidemia	NR	Hypoxic	CK: 4625 U/L<br>Creatinine: 2.4 mg/dL	NR	Rhabdomyolysis	None	Anticoagulation<br>Steroids<br>Remdesivir	Favorable
Khosla et al. 2020[18]	USA	Case series	64 M	DM2<br>HBP	NR	SOB<br>Nausea<br>Vomiting	CK: 3135 U/L	NR	Rhabdomyolysis	None	Azithromycin<br>Vitamin C<br>Vitamin D	Favorable
Kolkova et al. 2020[19]	Sweden	Case report	27 F	Obesity<br>Type-1 diabetes<br>Hypothyroidism	NR	Fever<br>Lower abdominal pain<br>Malaise<br>Headache<br>Cough<br>Dyspnea<br>Polyuria	Creatinine: 3.8 mg/dL	CT: bilateral diffuse ground-glass opacities	Rhabdomyolysis	AKI<br>Sepsis	Steroids<br>Mechanical ventilation<br>Betamethasone<br>Piperacillin/tazobactam	Favorable
Legrand et al. 2020[20]	USA	Case report	39 M	None	5 days	Chest pain<br>Dyspnea<br>Myalgias	CK: 17070 IU/L	CT: pleural and pericardial effusions, atelectasis	Rhabdomyolysis	Myopericarditis<br>Acute renal failure<br>Acute Hepatic Injury	Colchicine<br>Diuretics<br>Insulin<br>Oxygen	Favorable

Mah et al. 2020[21]	Singapore	Case report	32 M	Bilateral L5 nerve root radiculopathy	NR	Dry cough<br>Fever<br>Chest pain<br>Thigh pain<br>Difficulty walking	CK: 11071 U/L<br>AST: 81 U/L<br>Myoglobin: 208 µg/L	Normal	Rhabdomyolysis	None	IV fluids	Favorable
Meegada et al. 2020[22]	USA	Case report	19 M	Ulcerative colitis	NR	Diffuse abdominal pain<br>Fever	CK: 22000 U/L<br>ALT: 162 U/L<br>AST: 837 U/L	CT: fluid-filled colon	Rhabdomyolysis	None	IV fluids	Favorable
Mukherjee et al. 2020[23]	USA	Case report	49 M	HBP<br>DM	NR	Fever<br>Chills<br>Myalgia<br>Dyspnea	CK: 22740 U/L<br>CRP: 117 mg/L<br>AST: 470 U/L	CXR: bilateral patchy peripheral infiltrates	Rhabdomyolysis	AKI	IV fluids	Favorable
Munoz-Martinez et al. 2020[24]	USA	Case report	89 M	HBP<br>CAD<br>Heart failure	NR	Dyspnea<br>Fever<br>Cough<br>Fatigue<br>Difficulty breathing<br>Diarrhea	CK: 13025 U/L<br>Creatinine: 2.5 mg/dL	NR	Rhabdomyolysis	None	IV hydration<br>Supplemental oxygen	Favorable
Murillo et al. 2020[25]	Peru	Case report	48 M	None	NR	Myalgias<br>Asthenia<br>Dry cough<br>Fever<br>Dyspnea	CK: 10768 U/L<br>CK-MB:	CT: bilateral ground-glass opacifications	Rhabdomyolysis	HBP<br>AF	IV fluids<br>Azithromycin<br>Oseltamivir<br>Hydroxychloroquine	Favorable

							30.3 ng/mL<br>AST: 65 U/L<br>Pro-BNP: 304.2 pg/mL				Mechanical ventilation	
Rivas-García et al. 2020[26]	Spain	Case report	78 M	HBP<br>DM2	NR	Asthenia<br>Fever<br>Myalgias<br>Dark urine<br>Muscle weakness	CK: 11200 U/L<br>AST: 937 U/L<br>CRP: 131.2 mg/L<br>LDH: 972 U/L	NR	Rhabdomyolysis	NR	IV fluids<br>Hydroxychloroquine<br>Ritonavir/lopinavir	Favorable
Rosato et al. 2020[27]	Italy	Case report	58 M	HBP<br>Overweight	NR	Hypoxia<br>Tachypnea<br>Fever	CK: 3309 U/L<br>Creatinine: 3.5 mg/dL<br>Myoglobin: 3322 nmol/L	NR	Rhabdomyolysis	AKI	Mechanical ventilation<br>Continuous veno-venous hemofiltration	Favorable
Samies et al. 2020[28]	USA	Case report	16 M	Obesity<br>HBP<br>DM2<br>Obstructive sleep apnea	NR	Fever<br>Myalgia<br>Cough<br>Sore throat<br>Dark urine	CK: 426700 U/L<br>Creatinine: 12mg/dL	NR	Rhabdomyolysis	None	IV fluids	NR

							AST: 1316 U/L					
Sathappan et al. 2020[29]	USA	Case report	55 F	DM2<br>Deep vein thrombosis<br>Seizure disorder<br>Left hemiparesis	NR	Severe hyperglycemia<br>Acute change in physical mobility	CK: 43720 U/L<br>AST: 425 U/L<br>ALT: 86 U/L	CXR: subtle, patchy, left-mid lung zone opacity	Rhabdomyolysis	None	IV fluids<br>Insulin	Favorable
Shanbhag et al. 2020[30]	USA	Case report	19 M	Prior influenza-associated rhabdomyolysis	NR	Myalgia<br>Dry cough<br>Red-colored urine	CK: 694200 U/L<br>AST: 1014 U/L<br>LDH: 13950 U/L	None	Rhabdomyolysis	None	IV fluids	Favorable
Singh et al. 2020[31]	USA	Case series	67 M	HBP	NR	SOB<br>Fever	CK: 19773 U/L<br>CRP: 157.9 mg/L	CXR: bilateral hazy infiltrates<br>CT: ground-glass opacity and consolidation in the lungs bilaterally	Rhabdomyolysis	AKI	Azithromycin<br>Ceftriaxone<br>Hydroxychloroquine<br>Fluids	Death
Singh et al. 2020[31]	USA	Case series	39 M	HBP	NR	Muscle aches<br>Fever<br>SOB<br>Altered mental status	CK: 4330 U/L<br>Creatinine: 3.8 mg/dL<br>AST: 131 U/L	CXR: extensive confluent bilateral pulmonary infiltrates	Rhabdomyolysis	Cardiac arrest	NR	Death

Singh et al. 2020[31]	USA	Case series	43 M	End-stage renal disease	NR	Cough<br>SOB<br>Muscle aches<br>Fever	CK: 9793 U/L<br>Creatinine: 20 mg/dL<br>AST: 142 U/L	CXR: multifocal ill-defined parenchymal opacities bilaterally	Rhabdomyolysis	Cardiac arrest	Ceftriaxone<br>Hydroxychloroquine<br>Azithromycin	Death
Singh et al. 2020[31]	USA	Case series	70 M	None	NR	Cough<br>SOB	CK: 5008 U/L<br>AST: 179 U/L	CXR: bilateral hazy infiltrates throughout both lung fields	Rhabdomyolysis	Septic shock	IV methylprednisolone<br>Ceftriaxone<br>Azithromycin	Death
Singh et al. 2020[31]	USA	Case series	54 M	Asthma<br>DM<br>HBP<br>Obesity	NR	Cough<br>SOB<br>Fever	CK: 7337 U/L<br>AST: 161 U/L	NR	Rhabdomyolysis	NR	NR	Death
Singh et al. 2020[31]	USA	Case series	54 M	None	NR	Myalgias<br>Fever<br>Cough<br>SOB	CK: 3068 U/L<br>Creatinine: 11 mg/dL	NR	Rhabdomyolysis	NR	NR	Favorable
Singh et al. 2020[31]	USA	Case series	34 M	Obesity<br>Prediabetes	NR	Fever<br>Cough<br>SOB<br>Weakness	CK: 5454 U/L<br>AST: 100 U/L	NR	Rhabdomyolysis	NR	NR	Death
Singh et al. 2020[31]	USA	Case series	71 M	HBP<br>Seizures	NR	Fever<br>Cough<br>SOB	Creatinine: 4.1 mg/dL<br>AST: 128 U/L	NR	Rhabdomyolysis	NR	NR	Death

Singh et al. 2020[31]	USA	Case series	88 M	DM HBP	NR	Confusion	CK: 2628 U/L Creatinine: 2.2 mg/dL AST: 117 U/L	NR	Rhabdomyolysis	NR	NR	Death
Singh et al. 2020[31]	USA	Case series	56 M	HBP Prediabetes	NR	Fever Cough SOB	CK: 5388 U/L AST: 299 U/L	NR	Rhabdomyolysis	NR	NR	Favorable
Singh et al. 2020[31]	USA	Case series	57 M	None	NR	Cough	CK: 37524 U/L AST: 511 U/L	NR	Rhabdomyolysis	NR	NR	Death
Singh et al. 2020[31]	USA	Case series	64 M	None	NR	Myalgias Fever Cough SOB	CK: 6435 U/L AST: 113 U/L	NR	Rhabdomyolysis	NR	NR	Death
Singh et al. 2020[31]	USA	Case series	36 M	None	NR	Fever Cough SOB	CK: 5531 U/L AST: 177 U/L	NR	Rhabdomyolysis	NR	NR	Death
Singh et al. 2020[31]	USA	Case series	39 M	HBP	NR	Myalgias Fever Cough SOB	CK: 4330 U/L AST: 131 U/L	NR	Rhabdomyolysis	NR	NR	Death
Solís et al. 2020[32]	Mexico	Case report	46 M	CML	NR	Cough Fever Dyspnea	CK: 426700 U/L	CXR: bilateral and diffuse	Rhabdomyolysis	AKI	Antibiotics IV fluids CRRT	Death

						Muscle pain	Creatinine: 16.25 mg/dL<br>AST: 3565 U/L	ground-glass opacities				
Suwanwongse and Shabarek 2020[33]	USA	Case report	88 M	HBP<br>CKD<br>Heart failure<br>BPH<br>Osteoarthritis	NR	Bilateral thighs weakness and pain	CK: 13581 U/L<br>Creatinine: 1.4 mg/dL	CXR: left small pleural effusion	Rhabdomyolysis	AKI	IV crystalloids<br>IV furosemide<br>Hydroxychloroquine	Favorable
Taxbro et al. 2020[34]	Sweden	Case report	38 M	DM2<br>Gout<br>Obesity	NR	Fever<br>Myalgia<br>Abdominal pain<br>Breathlessness<br>Nausea<br>Vomiting	Creatinine: 3.1 mg/dL	CT: lungs showing extensive bilateral ground-glass opacities	Rhabdomyolysis	ARDS	CRRT	Favorable
Tafti et al. 2020[35]	USA	Case series	33 M	Sickle cell trait	NR	Collapse on a run<br>Back pain<br>Hemorrhage into his lumbosacral musculature<br>Fever	CK: 100000 U/L	CT: consolidation and ground glass opacities, cavitation<br>MRI: edema<br>US: anechoic fluid collection	Rhabdomyolysis	AKI<br>Pulmonary abscess<br>Methicillin-sensitive staph aureus pneumonia	Hemodialysis<br>Surgical evacuation	Favorable
Tram et al. 2020[36]	Belgium	Case report	15 M	None	NR	Vomiting<br>Diarrhea<br>Dark urine<br>Fatigue	Creatinine: 8.91 mg/dL	NR	Rhabdomyolysis	NR	Aggressive hydration<br>Sodium bicarbonate	Favorable

						Abdominal pain<br>Polyuria<br>Polydipsia	CK: 21876 U/L<br>AST: 319 U/L				
Valente-Acosta et al. 2020[37]	Mexico	Case report	71 M	BPH<br>Smoker	NR	Cough<br>Dyspnea<br>Fever<br>myalgia<br>Arthralgia<br>Weakness<br>Malaise	CK: 8720 U/L<br>LDH: 541 U/L<br>CRP: 26.9 mg/dL	CT: bilateral infiltrates with areas of consolidation and extensive ground-glass opacities	Rhabdomyolysis	None	Oxygen<br>Enoxaparin<br>Azithromycin<br>Ceftriaxone<br>Hydroxychloroquine<br>Lopinavir/ritonavir<br>Tocilizumab	Favorable
Wongkittichote et al. 2020[38]	USA	Case report	23 F	LCHADD	NR	Dry cough<br>Chest pain<br>Epigastric pain<br>Nausea	CK: 40600 U/mL<br>Creatinine: 2.25 mg/dL<br>AST: 1175 U/L	CT: small bilateral pleural effusions, ischemic nonocclusive enteritis	Rhabdomyolysis	AKI	IV fluids<br>Dextrose	Death
Zhang et al. 2020 [39]	USA	Case report	38 M	Gastroesophageal reflux	NR	Fever<br>Dyspnea<br>Myalgias	CK: 42 670 U/L<br>AST: 492 U/L	CXR: right upper and middle lobe consolidation with an air bronchogram	Rhabdomyolysis	Myositis	Cefepime<br>Azithromycin<br>Hydroxychloroquine<br>Doxycycline<br>IV fluids	Favorable
Zombori et al. 2020[40]	UK	Case report	17 F	Cornelia de Lange syndrome<br>Epilepsy	NR	Cough<br>SOB	CK: 7822 U/L /L	NR	Rhabdomyolysis	AKI<br>Cortical injury	Intubation	Unfavorable
Arai et al. 2021[41]	Japan	Case report	61 M	NR	1 day	Low blood pressure	CK: 8318 U/L	ECG: sinus tachycardia, ST elevation	Rhabdomyolysis	Hypovolemic shock	Corticoids<br>Dantrolene	Favorable

						Metabolic acidosis	CK-MB: 288 U/L Troponi n I: 9.63 ng/mL	Echo: LV hypertrophy with pericardial effusion			Renal replacement therapy	
Armanpoo r et al. 2021[42]	Iran	Case repo rt	10 M	None	NR	Fever<br>Dry cough<br>Muscle weakness<br>Pain in both legs	CK: 8000 U/L<br>AST: 117 U/L	CT: small ground-glass nodules	Rhabdomyo lysis	NR	Hydroxychloroq uine<br>Azithromycin	Favorable
Bach et al. 2021[43]	USA	Case repo rt	16 M	Morbid obesity<br>Anaphylaxis	NR	Myalgias	CK: 64560 U/L	CXR: poor expansion	Rhabdomyo lysis	NR	Normal saline<br>Acetaminophen<br>Methylprednisol one<br>Diphenhydramin e<br>Famotidine	Favorable
Bibbo 2021[44]	USA	Case repo rt	35 M	NR	NR	Fever<br>SOB<br>Cough	CK: 9633 U/L	CT: ground-glass appearance in all lung fields	Rhabdomyo lysis	Acute tubular necrosis	Surgical intervention	Favorable
Byler et al. 2021[45]	USA	Case repo rt	67 F	DM2<br>HBP<br>Hyperlipidem ia	1 month	Weakness	CK: 15085 U/L<br>Creatini ne: 10.9 mg/dL<br>AST: 749 U/L	CXR: extensive bilateral pulmonary opacities	Rhabdomyo lysis	Renal failure	Renal replacement therapy	Favorable
Cassim et al. 2021[46]	South Africa	Case repo rt	12 F	None	NR	Progressive weakness of the lower limbs<br>Myalgia	Creatini ne: 2.5 mg/dL	Normal	Rhabdomyo lysis	NR	Fluid resuscitation, RRT, IVIG<br>Methylprednisol one	Favorable

						Dyspnea	CK: 7134 U/L				Antibiotics	
Chetram et al. 2021[47]	USA	Case report	62 M	HBP<br>Obesity<br>DM2	NR	Malaise<br>Poor appetite<br>Hematuria<br>Fever	CK: 327 629 U/L<br>Creatinine: 4.9 mg/dL<br>AST: 1077 U/L	CXR: multifocal patchy airspace disease	Rhabdomyolysis	AKI	Lactated ringer<br>CRRT<br>Dexamethasone	Favorable
Chiba et al. 2021[48]	Japan	Case report	36 M	None	NR	Dyspnea<br>Fever<br>Cough<br>Fatigue	CK: 26046 U/L<br>Myoglobin: 3668 ng/mL	CXR: patchy bilateral opacities<br>CT: bilateral ground-glass opacities	Rhabdomyolysis	Malignant hyperthermia	Antibiotics<br>Antipyretics<br>Steroids<br>Antivirals<br>IV hydration<br>Dantrolene	Favorable
Desai et al. 2021[49]	USA	Case report	21 M	None	NR	Bilateral arm pain	CK: 67873 U/L<br>AST: 219 U/L<br>ALT: 219 U/L	US: mild splenomegaly	Rhabdomyolysis	Neutropenia	IV fluids	Favorable
Egoryan et al. 2021[50]	USA	Case report	50 M	DM2<br>HBP<br>Hyperlipidemia	NR	Dark urine<br>Decreased urine output<br>Constipation<br>Fever	CK: 359910 U/L<br>Creatinine: 2.3 mg/dL<br>AST: 2222 U/L<br>ALT: 432 U/L	CT: bibasilar ground-glass opacities	Rhabdomyolysis	AKI<br>Liver injury	Aggressive IV fluid therapy<br>Oxygen<br>Dexamethasone	Favorable

Fabi et al. 2021[51]	Italy	Case report	6 F	None	NR	Abdominal pain<br>Chest pain<br>Asthenia<br>Fever<br>Severe myalgia<br>Oliguria	CK: 3392 U/L<br>Creatinine: 1.1 mg/dL	CT: pneumonia in the right lower lobe with moderate pleural effusion<br>US: mild free abdominal effusion	Rhabdomyolysis	Multisystem inflammatory syndrome	Hyperhydration<br>IVIG<br>Steroids	Favorable
Fadel et al. 2021[52]	USA	Case report	33 M	None	NR	Myalgias<br>Headache<br>Loss of taste<br>Dyspnea<br>Hematuria	AST: 1 835 U/L<br>CK: 362 445 U/L	CXR: left lower lobe infiltrate<br>CT: infiltrates involving the bilateral lower lobe	Rhabdomyolysis	NR	IVF with lactate Ringer<br>Dexamethasone<br>Azithromycin<br>Rocephin<br>Enoxaparin<br>Fluid management	Favorable
Fadiran 2021[53]	USA	Case report	78 F	Hypothyroidism<br>Dyslipidemia	NR	Developed profound	CK: 10650 U/L	CXR: ill-defined hazy ground-glass opacities	Rhabdomyolysis	Pancytopenia<br>Myocarditis	Ringer's lactate<br>Remdesivir<br>Dexamethasone	Favorable
Farouji et al. 2021[54]	USA	Case report	21 M	None	7 days	Fever<br>Cough<br>SOB	CK: 53886 U/L	CXR: infiltrates in the right lower hemithorax	Rhabdomyolysis	NR	IV infusion	Favorable
Flato et al. 2021[55]	Brazil	Case report	18 F	Obesity	NR	Myalgia<br>Dry cough<br>Fever<br>Dyspnea<br>Tachypnea<br>Hypoxemia	CK: 18263 U/L<br>AST: 202 U/L	CT: 50% ground-glass pattern	Rhabdomyolysis	Organ dysfunction	Oxygen therapy<br>Antibiotics<br>Olsetamivir	Death

Fujita et al. 2021[56]	Japan	Case report	19 F	None	NR	Fever<br>Cough<br>Fatigue<br>Muscle pain	CK: 55613 U/L<br>AST: 1013 U/L	CT: patchy infiltrative shadow in the left lower lobe	Rhabdomyolysis	NR	IV infusion	Favorable
Gilpin et al. 2021[57]	USA	Case report	16 M	Asthma	NR	Dark urine<br>Bilateral pain in shoulders and thighs	CK: 392488 U/L<br>AST: 662 U/L	NR	Rhabdomyolysis	None	Glucocorticoids	Favorable
Heidarpour et al. 2021[58]	Iran	Case series	22 M	None	NR	Muscle spasms and pain<br>Diarrhea	CK: 1840 U/L<br>AST: 261 U/L<br>ALT: 110 U/L	CT: multifocal peribronchial consolidation and diffuse ground-glass opacities	Rhabdomyolysis	Seizure	IV fluids	Death
Heidarpour et al. 2021[58]	Iran	Case series	36 M	Psychosis<br>Drug use	NR	Polyuria<br>Polydipsia<br>Nausea<br>Vomiting<br>Dyspnea<br>SOB	CK: 5130 U/L<br>Creatinine: 5.3 mg/dL	CT: bilateral and peripheral ground-glass pulmonary opacities.	Rhabdomyolysis	NR	Hemodialysis<br>Insulin	Favorable
Heidarpour et al. 2021[58]	Iran	Case series	47 F	DM2<br>HBP<br>Hypothyroidism<br>Asthma	NR	Fever<br>Cough<br>Dyspnea	CK: 16480 U/L<br>Creatinine: 1.7 mg/dL<br>AST: 951 U/L<br>ALT: 874 U/L	CT: Multifocal multilobular consolidation and ground-glass opacities	Rhabdomyolysis	ARDS	Serum therapy<br>Oxygen	Death

Hookey et al. 2021[59]	USA	Case series	64 M	DM2<br>HBP<br>Heart failure<br>CKD	NR	Progressive malaise<br>Weakness<br>Abdominal pain<br>Vomiting<br>Loss of appetite	CK: 19525 U/L<br>AST: 1104 U/L<br>ALT: 189 U/L	CT: multifocal ground glass opacities in the lung bases<br>Tc-99m: widespread radiotracer uptake	Rhabdomyolysis	NR	IV fluids<br>Steroids<br>Dialysis	Favorable
Hookey et al. 2021[59]	USA	Case series	34 M	None	NR	Dark urine<br>Decreased urine output<br>Body aches<br>Fever<br>Chills	CK: 200000 U/L<br>AST: 2420 U/L<br>ALT: 368 U/L	Tc-99m: widespread radiotracer uptake<br>US: renal disease	Rhabdomyolysis	NR	IV fluids<br>Steroids<br>Dialysis	Favorable
Lamzouri et al. 2021[60]	Morocco	Case report	63 -	None	NR	Myalgia<br>Fever	Creatinine: 2.6 mg/dL<br>CK: 7098 UI/L<br>AST: 538 UI/L	CT: ground-glass opacities in the periphery of both lungs	Rhabdomyolysis	None	Heparin<br>Saline dose<br>Dexamethasone	Favorable
Li et al. 2021[61]	UK	Case report	22 F	None	NR	Sore throat<br>Myalgia<br>Anuria	CK: 764470 U/L<br>Creatinine: 8.1 mg/dL<br>ALT: 442 U/L	NR	Rhabdomyolysis	NR	Renal replacement therapy<br>Continuous haemofiltration	Favorable

Mahmood et al. 2021[62]	USA	Case report	81 F	HBP<br>DM2<br>Hyperlipidemia<br>CKD	NR	Generalized myalgia<br>Weakness<br>Malaise<br>Cough<br>Dyspnea<br>Loss of appetite	CK: 6467 U/L<br>AST: 45 U/L	CXR: mild mixed patchy interstitial and alveolar infiltrates	Rhabdomyolysis	NR	IV crystalloid	Favorable
Mughal et al. 2021[63]	USA	Case report	39 M	None	NR	Muscle weakness<br>Fatigue	CK: 36064 U/L	MRI: heterogenous enhancement of the iliopsoas musculature	Rhabdomyolysis	Acute viral myositis	IVIG<br>IV fluids<br>Steroids	Favorable
Patel et al. 2021[64]	USA	Case report	60 M	CKD<br>Asthma	NR	Generalized fatigue<br>Myalgia<br>Mild cough	CK: >40000 U/L<br>AST: 930 UI/L<br>ALT: 117 UI/L	CT: diffuse ground-glass opacities in both lungs	Rhabdomyolysis	ARDS<br>Multiorgan failure<br>Cardiac arrest	Ringer’s lactate<br>Oxygen supplementation<br>Antibiotics<br>Dexamethasone	Death
Pérez et al. 2021[65]	Chile	Case report	35 F	Overweight	NR	Fever<br>Polymyalgia<br>Dyspnea	CK: 269328 U/L	CT: extensive multifocal pneumonia<br>MR: diffuse rhabdomyolysis	Rhabdomyolysis	AKI	Fluids<br>Invasive mechanical ventilation	Favorable
Riccardi et al. 2021[66]	USA	Case report	68 M	HBP<br>Prediabetes<br>CKD	2 days	Fever<br>Tachycardia<br>Hypotension<br>Hypoxia<br>Tachypnea	Creatinine: 3.3 mg/dL<br>AST: 251 U/L	CXR: bilateral patchy opacities consistent	Rhabdomyolysis	NR	Volume resuscitation<br>Dexamethasone	Favorable

							CK : 16716 U/L					
Toro et al. 2021[67]	Chile	Case report	37 M	None	NR	Myalgias<br>Nausea<br>Epigastric pain radiating to the back	CK: 62768 U/L<br>Creatinine: 2.1 mg/dL	CXR: Bilateral alveolar filling opacities<br>CT: frosted glass peripheral spotlights	Rhabdomyolysis	None	Hydration<br>Sodium bicarbonate<br>Ceftriaxone<br>Azithromycin	Favorable
Vasquez-Garagatti et al. 2021[68]	USA	Case report	62 F	HBP<br>Hypothyroidism<br>Asthma	NR	Myalgia<br>Weakness<br>Malaise	CK: 19629 U/L<br>AST: 441 U/L<br>ALT: 200 U/L	NR	Rhabdomyolysis	None	IV hydration<br>Doxycycline<br>Enoxaparin	Favorable
Veyseh et al. 2021[69]	USA	Case report	57 F	HBP	NR	Dyspnea<br>Muscle weakness	CK: 15000 U/L	MRI: inflammation of the thigh muscles	Rhabdomyolysis	None	Prednisone	Favorable
Wu et al. 2021[70]	Taiwan	Case report	74 F	HBP<br>DM2	NR	Fever<br>Dyspnea	CK: 1643 U/L<br>Creatinine: 1.3 mg/dL	CXR: Interstitial infiltrates and opacities over both lungs	Rhabdomyolysis	NR	Hydroxychloroquine<br>Lopinavir/ritonavir<br>Azithromycin<br>IVIG Baricitinib	Favorable
Abdelmottaleb et al. 2022[71]	USA	Case report	42 F	None	NR	Generalized weakness<br>Myalgia<br>Epigastric pain	CK: 25000 U/L	None	Rhabdomyolysis	Myopericarditis with pericardial effusion<br>Cardiac tamponade	Colchicine<br>Ibuprofen<br>IV fluids	Favorable

Banamah et al. 2022[72]	USA	Case report	58 F	None	NR	Lethargy<br>Lower limb weakness<br>Dark urine	CK: 9202 U/L<br>Creatinine: 8.6 mg/dL	CT: diffuse subcutaneous edema bilaterally	Rhabdomyolysis	NR	IV fluids	Favorable
Bawor et al. 2022[73]	UK	Case report	64 F	HBP<br>Episodes of rhabdomyolysis	NR	Bilateral thigh pain<br>Dark urine<br>Lethargy<br>Cough<br>Taste disturbance	CK: 119301 UI/L	None	Rhabdomyolysis	None	IV fluids<br>Enoxaparin<br>Paracetamol	Favorable
Donati et al. 2022[74]	Italy	Case report	38 M	None	NR	Dyspnea<br>Lower back pain<br>Lower limbs hyposthenia<br>Pharyngotympanic	Creatinine: 11.2 mg/dL<br>CK: >20 000 U/L<br>AST: 2953U/L	CT: peripheral ground-glass opacities	Rhabdomyolysis	AKI	Hemodialysis<br>Furosemide	Favorable
Freund et al. 2022[75]	Israel	Case report	91 M	HBP<br>Dyslipidemia<br>Atril fibrillation<br>CKD	NR	Lower limb weakness<br>SOB	CK: 8575 U/L	CXR: enlarged heart silhouette	Rhabdomyolysis	NR	Nasal cannula<br>Dexamethasone	Death
Jahnke et al. 2022[76]	Brazil	Case report	69 F	HBP<br>AMI<br>Cardiopathy	NR	Dyspnea<br>Diarrhea<br>SOB	AST: 141 U/L<br>Creatinine: 4.8 mg/dL<br>CK: 4415 U/L	NR	Rhabdomyolysis	Worsening of kidney function	IV fluids<br>Diuretics<br>Insulin<br>Antipyretic<br>Antibiotic<br>Analgesic	Death

Kar et al. 2022[77]	USA	Case report	50 F	Hypothyroidism	NR	Fatigue Nausea Vomiting Dyspnea Bilateral lower weakness Tightness	CK: 447800 U/L AST: 6053 U/L	CT: bilateral peripheral patchy ground-glass opacification	Rhabdomyolysis	NR	IV fluids Empiric antibiotics Hydrocortisone IV levothyroxine	Favorable
Kermond et al. 2022[78]	Australia	Case report	15 F	Sickle cell trait	NR	Cough Fever Myalgia Poor oral intake Tachycardia Tachypnea	CK: 746352 U/L Creatinine: 5.85 mg/dL	NR	Rhabdomyolysis	NR	IV fluid Furosemide	Favorable
Kontou et al. 2022[79]	Greece	Case report	10 F	None	NR	Fever Myalgia Limping	CK: 13147 U/L AST: 288 U/L	Normal	Rhabdomyolysis	None	IV fluids	Favorable
Mariano and MacLaren 2022[80]	USA	Case report	28 F	Asthma Tobacco abuse	NR	Fever cough SOB Myalgia Weakness Dark urine	CK: >40000 U/L	CT: consolidation in the right middle and right lower lobes	Rhabdomyolysis	NR	Remdesivir Mechanical ventilation	Death
Merbouh et al. 2022[81]	Morocco	Case report	52 F	HBP	NR	Myalgia Fever	CK: 15000 UI/L Creatinine: 1.46 mg/dL	NR	Rhabdomyolysis	None	Saline Oxygen Norepinephrine Antibiotics Hydrocortisone	Favorable
Miranda et al. 2022[82]	Brazil	Case report	31 F	COVID-19 infection	NR	Ankle pain Edema Choluria	Creatinine: 8.5 mg/dL	NR	Rhabdomyolysis	NR	IV fluids	Favorable

						Dyspnea	CK: 120053 U/L<br>AST: 4657U/L					
Mittal et al. 2022[83]	USA	Case report	34 M	HBP	3 weeks	Decreased mental status<br>Asterixis	CK: 289500 mcg/l<br>Creatinine: 2.9 mg/dL<br>AST: 1420 U/L	NR	Rhabdomyolysis	Acute tubular necrosis	Hemodialysis<br>IV fluids	Favorable
Rambabova-Bushljetik et al. 2022 [84]	Macedonia	Case report	18 M	CPT II deficiency	3 weeks	Myalgia<br>Muscular weakness<br>Dark urine	AST: 2058 U/L<br>CK: 102410 U/L	Normal	Rhabdomyolysis	NR	Extensive fluid replacement	Favorable
Takada et al. 2022[85]	Japan	Case report	52 M	Schizophrenia	NR	Back pain<br>Tremor<br>Weakness	CK: 41539 U/L<br>CK-MB: 109 U/L<br>AST: 769 U/L	CT: bilateral peripheral ground-glass opacity	Rhabdomyolysis	NR	IV fluids<br>Remdesivir<br>Dexamethasone<br>Oxygenation	Favorable
Tuan et al. 2022[86]	USA	Case report	67 M	AF<br>Critical aortic stenosis<br>CAD<br>CABG	NR	Fatigue<br>Myalgias<br>Nausea<br>Vomiting<br>Diarrhea<br>Anuria	Creatinine: 9.55 mg/dL<br>CK: 521 U/l	CT: colitis, horseshoe kidney, ground glass and nodular lesion in the	Rhabdomyolysis	AKI	IV fluids<br>Piperacillin–tazobactam<br>Metronidazole<br>Ceftriaxone<br>Hemodialysis	Favorable

				Severe emphysematous COPD				right middle lobe				
Wu et al. 2022[87]	Taiwan	Case series	6 M	NR	NR	Calf pain<br>Fever	CK: 1596 U/L<br>CK-MB: 19.8 ng/mL	NR	Rhabdomyolysis	None	Normal saline hydration<br>Ibuprofen<br>Acetaminofen	Favorable
Wu et al. 2022[87]	Taiwan	Case series	10 F	NR	NR	Calf pain<br>Fever	CK: 3180 U/L<br>CK-MB: 66.8 ng/mL<br>AST: 184 U/L	NR	Rhabdomyolysis	None	Normal saline hydration<br>Ibuprofen<br>Acetaminofen	Favorable
Wu et al. 2022[87]	Taiwan	Case series	10 M	NR	NR	Calf pain<br>Fever	CK: 1543 U/L<br>CK-MB: 47.5 ng/mL<br>AST: 68 U/L	NR	Rhabdomyolysis	None	Normal saline hydration<br>Ibuprofen<br>Acetaminofen	Favorable
Wu et al. 2022[87]	Taiwan	Case series	6 F	NR	NR	Calf pain<br>Fever	CK: 6937 U/L<br>CK-MB: 91 ng/mL<br>AST: 170 U/L	NR	Rhabdomyolysis	None	Normal saline hydration<br>Ibuprofen<br>Acetaminofen	Favorable

Wu et al. 2022[87]	Taiwan	Case series	8 M	NR	NR	Calf pain<br>Fever	CK: 1614 U/L<br>CK-MB: 20.9 ng/mL<br>AST: 66 U/L	NR	Rhabdomyolysis	None	Normal saline hydration<br>Ibuprofen<br>Acetaminofen	Favorable
Wu et al. 2022[87]	Taiwan	Case series	7 M	NR	NR	Calf pain<br>Fever	CK: 1892 U/L<br>CK-MB: 30.1 ng/mL<br>AST: 65 U/L	NR	Rhabdomyolysis	None	Normal saline hydration<br>Ibuprofen<br>Acetaminofen	Favorable
Wu et al. 2022[87]	Taiwan	Case series	6 M	NR	NR	Calf pain<br>Fever	CK: 1346 U/L<br>CK-MB: 18.2 ng/mL<br>AST: 119 U/L	NR	Rhabdomyolysis	None	Normal saline hydration<br>Ibuprofen<br>Acetaminofen	Favorable
Wu et al. 2022[87]	Taiwan	Case series	5 M	NR	NR	Calf pain<br>Fever	CK: 1475 U/L<br>CK-MB: 19.2 ng/mL<br>AST: 60 U/L	NR	Rhabdomyolysis	None	Normal saline hydration<br>Ibuprofen<br>Acetaminofen	Favorable

Yu et al. 2022[88]	USA	Case report	19 M	Asthma<br>Obesity<br>Myocardial infarction	NR	Myalgia<br>Diaphoresis<br>Dark urine	CK: 346695 U/L<br>AST: 1234 U/L<br>Creatinine: 1.8 mg/dL	Normal	Rhabdomyolysis	NR	IV fluids<br>Sodium bicarbonate<br>Rasburicase	Favorable
Geeting et al. 2023[89]	USA	Case report	15 M	Obesity	NR	Emesis<br>Diarrhea<br>Weakness<br>Myalgias<br>Cough	CK: 584886 U/L<br>CK-MB: 17.3 ng/mL<br>AST: 2086 U/L	ECG: peaked T-waves	Rhabdomyolysis	NR	IV fluids<br>Insulin<br>CRRT<br>Sodium bicarbonate	Favorable
Hassan et al. 2023[90]	USA	Case report	89 M	Non-ischemic cardiomyopathy<br>HBP<br>Obesity<br>CKD	NR	Chest pain<br>SOB<br>Dizziness<br>Loss of consciousness	CK: 15980 U/L<br>Creatinine: 1.68 mg/dL	ECG: atrioventricular dual-paced rhythm	Rhabdomyolysis	NR	Antiviral<br>Antibiotics	Favorable
Khalayli et al. 2023[91]	Syria	Case report	48 F	None	NR	Myalgia<br>Arthralgia<br>Fever	CRP: 36 mg/dL<br>CK: 542310 UI/L<br>AST: 1246 U/L<br>ALT: 986 U/L	CT: mild pulmonary infiltrations	Rhabdomyolysis	Cardiac arrest	Epinephrine<br>Azithromycin<br>Methyl prednisone<br>Enoxaparin<br>Mechanically ventilated	Death

Lu et al. 2023[92]	China	Case report	85 M	CKD<br>HBP	NR	Fatigue<br>Muscle pain and weakness<br>Back pain<br>Difficulty walking	CK: 32492.9 U/L<br>Creatinine: 896.7 µmol/L<br>AST: 421.6 U/L	CT: muscle atrophy, widened muscle spaces and increased fat density shadows	Rhabdomyolysis	NR	CRRT	Favorable
Matsuki et al. 2023[93]	Japan	Case report	62 M	None	3 days	Anemia<br>Liver dysfunction<br>Confusion	CK: 9498 U/L<br>Creatinine: 1.25 mg/dL<br>AST: 5398 U/L	CT: bronchopneumonia in the left upper lobe of the lung	Rhabdomyolysis	None	Antibiotics<br>FFP	Favorable
Sotirios et al. 2023[94]	Greece	Case report	62 M	Coronary artery bypass<br>HBP	NR	Fever<br>Dry cough<br>Dyspnea	CK: 200 U/L	CXR: bilateral opacities	Rhabdomyolysis	NR	Furosemide<br>Midazolam<br>Propofol<br>Neuromuscular blocking agents<br>Vasopressors	Favorable
Dye and Lantz 2024[95]	USA	Case report	22 M	None	NR	Fever<br>Cough<br>Chills<br>Nausea<br>Myalgias<br>Dark urine	AST: 1553 U/L<br>CK: 1658 U/L	Normal	Rhabdomyolysis	NR	Ketorolac<br>Crystalloids	Favorable
Nayak et al. 2024[96]	India	Case report	50 M	Nephrotic syndrome<br>CKD<br>Hypothyroidism	NR	Edema	CK: > 22 000 U/L	Normal	Rhabdomyolysis	NR	IV fluids<br>IV 25% albumin<br>Diuretics	Favorable

				Epilepsy<br>Bipolar disorder			Creatinine: 3.5 mg/dL<br>AST: 1768 U/L					
Okubo et al. 2024[97]	Japan	Case report	28 M	Panhypopituitarism	NR	Fatigue	CK: 749 U/L<br>AST: 320 U/L	CT: infiltrates in the right lung	Rhabdomyolysis	NR	IV fluids<br>Hydrocortisone<br>Antibiotics	Favorable
Reddy et al. 2024[98]	USA	Case report	56 F	Hepatitis A<br>Uterine fibroids	NR	Dyspnea<br>Nasal congestion<br>Fever<br>Generalized malaise<br>Bilateral severe thigh muscle contractions<br>Dark urine	CK: >50000 U/L<br>AST: 1128 U/L	MRI of bilateral lower extremities: mild myositis	Rhabdomyolysis	Polymyositis	IV normal saline<br>Methylprednisolone	Favorable
Sripathy et al. 2024[99]	India	Case report	31 M	β-thalassemia trait	4 days	Fever<br>Chills<br>Cough<br>Sore throat	CK: 77909 U/L<br>AST: 605 U/L	Normal	Rhabdomyolysis	None	Dexamethasone<br>Antibiotics<br>Acetaminophen<br>Enoxaparin	Unfavorable
Yuan et al. 2024[100]	China	Case report	53 M	None	NR	Fever Cough<br>Dark urine	CK: 39580 U/L<br>AST: 469.5 U/L	CT: double lung viral pneumonia	Rhabdomyolysis	None	Analgesics<br>Ventilator-assisted breathing<br>Antiviral and anti-infection medications	Favorable

ACLS: advanced cardiovascular life support; AF: atrial fibrillation; AKI: acute kidney injury; ALT: alanine transaminase; AMI: acute myocardial infarction; ARDS: acute respiratory distress syndrome; AST: aspartate transaminase; BPH: benign prostatic hypertrophy; CABG: coronary artery bypass graft; CAD: coronary artery disease; CK:

creatine kinase; CKD: chronic kidney disease; CK-MB: creatine kinase-MB; CML: chronic myeloid leukemia; CMR: Cardiac magnetic resonance imaging; COPD: chronic obstructive pulmonary disease; CPT II: Carnitine palmitoyltransferase II; CRP: c-reactive protein; CRRT: continuous renal replacement therapy; CT: computed tomography; Echo: echocardiography; ESRD: End-Stage Renal Disease; F: female; FFP: fresh frozen plasma; HBP: high blood pressure; HIV: human immunodeficiency virus; JVD: jugular venous distention; LCHADD: Long-chain fatty-acyl CoA dehydrogenase deficiency; LV: left ventricular; M: male; MR: magnetic resonance imaging; NR: not reported; Pro-BNP: pro-B-type natriuretic peptide; RRT: renal replacement therapy; SOB: shortness of breath; Tc-99 m: Technitium-99 m bone scan.

REFERENCES

1. Alrubaye, R.; Choudhary, H. Severe Rhabdomyolysis in a 35-Year-Old Woman with COVID-19 Due to SARS-CoV-2 Infection: A Case Report. *Am J Case Rep* **2020**, *21*, e926733-1-e926733-5, doi:10.12659/AJCR.926733.
2. Anklesaria, Z.; Frankman, J.; Gordin, J.; Zhan, J.; Liu, A.K. Fatal Rhabdomyolysis in a COVID-19 Patient on Rosuvastatin. *Cureus 12*, e11186, doi:10.7759/cureus.11186.
3. Aria, A.; Forouharnejad, K.; Mortazavi, M.; Omidi, A.; Askari, M.; Ghadimi, K.; Mashinchi-Asl, N. COVID 19 with Neurological Symptoms, Rhabdomyolysis and Brain Death: A Case Report. *Am J Clin Exp Immunol* **2020**, *9*, 114–117.
4. Anwar, H.; Al Lawati, A. Adolescent COVID-19-Associated Fatal Rhabdomyolysis. *J Prim Care Community Health* **2020**, *11*, 2150132720985641, doi:10.1177/2150132720985641.
5. Borku Uysal, B.; Ikitimur, H.; Yavuzer, S.; Islamoglu, M.S.; Cengiz, M. Case Report: A COVID-19 Patient Presenting with Mild Rhabdomyolysis. *Am J Trop Med Hyg* **2020**, *103*, 847–850, doi:10.4269/ajtmh.20-0583.
6. Buckholz, A.P.; Kaplan, A.; Rosenblatt, R.E.; Wan, D. Clinical Characteristics, Diagnosis, and Outcomes of 6 Patients With COVID-19 Infection and Rhabdomyolysis. *Mayo Clin Proc* **2020**, *95*, 2557–2559, doi:10.1016/j.mayocp.2020.09.005.
7. Case, R.; Ramaniuk, A.; Martin, P.; Simpson, P.J.; Harden, C.; Ataya, A. Systemic Capillary Leak Syndrome Secondary to Coronavirus Disease 2019. *Chest* **2020**, *158*, e267–e268, doi:10.1016/j.chest.2020.06.049.
8. Chan, K.H.; Farouji, I.; Abu Hanoud, A.; Slim, J. Weakness and Elevated Creatinine Kinase as the Initial Presentation of Coronavirus Disease 2019 (COVID-19). *Am J Emerg Med* **2020**, *38*, 1548.e1-1548.e3, doi:10.1016/j.ajem.2020.05.015.
9. Chedid, N.R.; Udit, S.; Solhjou, Z.; Patanwala, M.Y.; Sheridan, A.M.; Barkoudah, E. COVID-19 and Rhabdomyolysis. *J Gen Intern Med* **2020**, *35*, 3087–3090, doi:10.1007/s11606-020-06039-y.
10. Chong, W.H.; Saha, B. Severe Acute Respiratory Syndrome Coronavirus 2 (SARS-CoV-2) Associated with Rhabdomyolysis and Acute Kidney Injury (AKI). *Am J Med Sci* **2020**, *360*, 738–739, doi:10.1016/j.amjms.2020.07.032.
11. Cunha, M.; Pinho, I.; Lopes, M.; Trigueiros, F.; Braz, S.; Medeiros, F. A Case of Corticosteroid-Responsive SARS-CoV-2 Related Massive Rhabdomyolysis. *IDCases* **2020**, *22*, e00946, doi:10.1016/j.idcr.2020.e00946.
12. Foster, A.; Khan, Z.; Siddiqui, A.; Singh, S.; Atere, M.; Nfonoyim, J.M. It's Complicated: A Case Report on a COVID-19-Positive HIV Patient Presenting with Rhabdomyolysis and Acute Kidney Injury. *SAGE Open Med Case Rep* **2020**, *8*, 2050313X20965423, doi:10.1177/2050313X20965423.
13. Fujimaru, T.; Shimada, K.; Hamada, T.; Watanabe, K.; Ito, Y.; Nagahama, M.; Taki, F.; Isokawa, S.; Hifumi, T.; Otani, N.; et al. Development of Acute Kidney Injury with Massive Granular Casts and Microscopic Hematuria in Patients with COVID-19: Two Case Presentations with Literature Review. *Ren Replace Ther* **2020**, *6*, 59, doi:10.1186/s41100-020-00308-6.
14. Gefen, A.M.; Palumbo, N.; Nathan, S.K.; Singer, P.S.; Castellanos-Reyes, L.J.; Sethna, C.B. Pediatric COVID-19-Associated Rhabdomyolysis: A Case Report. *Pediatr Nephrol* **2020**, *35*, 1517–1520, doi:10.1007/s00467-020-04617-0.
15. Husain, R.; Corcuera-Solano, I.; Dayan, E.; Jacobi, A.H.; Huang, M. Rhabdomyolysis as a Manifestation of a Severe Case of COVID-19: A Case Report. *Radiol Case Rep* **2020**, *15*, 1633–1637, doi:10.1016/j.radcr.2020.07.003.

16. Jin, M.; Tong, Q. Rhabdomyolysis as Potential Late Complication Associated with COVID-19. *Emerg Infect Dis* **2020**, *26*, 1618–1620, doi:10.3201/eid2607.200445.
17. Kajani, R.; Apramian, A.; Vega, A.; Ubhayakar, N.; Xu, P.; Liu, A. Neuroleptic Malignant Syndrome in a COVID-19 Patient. *Brain Behav Immun* **2020**, *88*, 28–29, doi:10.1016/j.bbi.2020.05.042.
18. Khosla, S.G.; Nylen, E.S.; Khosla, R. Rhabdomyolysis in Patients Hospitalized With COVID-19 Infection: Five Case Series. *J Investig Med High Impact Case Rep* **2020**, *8*, 2324709620984603, doi:10.1177/2324709620984603.
19. Kolkova, Z.; Bjurström, M.F.; Länsberg, J.-K.; Svedas, E.; Hamer, M.A.; Hansson, S.R.; Herbst, A.; Zaigham, M. Obstetric and Intensive-Care Strategies in a High-Risk Pregnancy with Critical Respiratory Failure Due to COVID-19: A Case Report. *Case Rep Womens Health* **2020**, *27*, e00240, doi:10.1016/j.crwh.2020.e00240.
20. Legrand, F.; Chong-Nguyen, C.; Ghanem, N. Myopericarditis, Rhabdomyolysis, and Acute Hepatic Injury: Sole Expression of a SARS-CoV-2 Infection. *Circ Cardiovasc Imaging* **2020**, *13*, e010907, doi:10.1161/CIRCIMAGING.120.010907.
21. Mah, T.J.; Lum, Y.H.; Fan, B.E. Coronavirus Disease 2019 Presenting with Rhabdomyolysis. *Proceedings of Singapore Healthcare* **2021**, *30*, 68–70, doi:10.1177/2010105820943911.
22. Meegada, S.; Muppidi, V.; Wilkinson, D.C.; Siddamreddy, S.; Katta, S.K. Coronavirus Disease 2019-Induced Rhabdomyolysis. *Cureus 12*, e10123, doi:10.7759/cureus.10123.
23. Mukherjee, A.; Ghosh, R.; Aftab, G. Rhabdomyolysis in a Patient With Coronavirus Disease 2019. *Cureus 12*, e8956, doi:10.7759/cureus.8956.
24. Munoz-Martinez, A.; Waheed, M.A.; Jhaveri, K.D.; Rojas-Marte, G. COVID-19 Related Rhabdomyolysis. *Journal of Clinical Nephrology* **2020**, *4*, 065–069, doi:10.29328/journal.jcn.1001061.
25. Murillo, F.; Ramos, G.; del Pozo, J.L.; Valdez, L.M.; Zagaceta, J. SARS-CoV-2 Infection with Associated Rhabdomyolysis and Probable Myocarditis. *Eur J Case Rep Intern Med* **2020**, *7*, 001867, doi:10.12890/2020_001867.
26. Rivas-García, S.; Bernal, J.; Bachiller-Corral, J. Rhabdomyolysis as the Main Manifestation of Coronavirus Disease 2019. *Rheumatology (Oxford)* **2020**, *59*, 2174–2176, doi:10.1093/rheumatology/keaa351.
27. Rosato, C.; Bolondi, G.; Russo, E.; Oliva, A.; Scognamiglio, G.; Mambelli, E.; Longoni, M.; Rossi, G.; Agnoletti, V. Clinical, Electromyographical, Histopathological Characteristics of COVID-19 Related Rhabdomyolysis. *SAGE Open Med Case Rep* **2020**, *8*, 2050313X20983132, doi:10.1177/2050313X20983132.
28. Samies, N.L.; Pinninti, S.; James, S.H. Rhabdomyolysis and Acute Renal Failure in an Adolescent With Coronavirus Disease 2019. *J Pediatric Infect Dis Soc* **2020**, piaa083, doi:10.1093/jpids/piaa083.
29. Sathappan, A.; Wachs, D.; Morgenstein, C. A Potential Complication of COVID-19: A Case of Non-Traumatic Rhabdomyolisis. *J Med Cases* **2020**, *11*, 296–298, doi:10.14740/jmc3546.
30. Shanbhag, A.; Manaktala, P.S.; Rizvi, H.; Frey, K.; Narayanan, R. COVID-19 Presenting as Severe Rhabdomyolysis With Normal Renal Function. *Cureus 12*, e9556, doi:10.7759/cureus.9556.
31. Singh, B.; Kaur, P.; Mechineni, A.; Maroules, M. Rhabdomyolysis in COVID-19: Report of Four Cases. *Cureus* **2020**, doi:10.7759/cureus.10686.

32. Solís, J.G.; Esquivel Pineda, A.; Alberti Minutti, P.; Albarrán Sánchez, A. Case Report: Rhabdomyolysis in a Patient with COVID-19: A Proposed Diagnostic-Therapeutic Algorithm. *Am J Trop Med Hyg* **2020**, *103*, 1158–1161, doi:10.4269/ajtmh.20-0692.
33. Suwanwongse, K.; Shabarek, N. Rhabdomyolysis as a Presentation of 2019 Novel Coronavirus Disease. *Cureus 12*, e7561, doi:10.7759/cureus.7561.
34. Taxbro, K.; Kahlow, H.; Wulcan, H.; Fornarve, A. Rhabdomyolysis and Acute Kidney Injury in Severe COVID-19 Infection. *BMJ Case Rep* **2020**, *13*, e237616, doi:10.1136/bcr-2020-237616.
35. Tafti, D.; Kluckman, M.; Dearborn, M.C.; Hunninghake, J.; Clayton, S. COVID-19 in Patients With Hematologic-Oncologic Risk Factors: Complications in Three Patients. *Cureus 12*, e12064, doi:10.7759/cureus.12064.
36. Tram, N.; Chiodini, B.; Montesinos, I.; Vicinanza, A.; Beretta-Piccoli, X.; Gubbelmans, N.; Demey, M.; Genis, N.; Tilmanne, A.; Smeesters, P.R.; et al. Rhabdomyolysis and Acute Kidney Injury as Leading COVID-19 Presentation in an Adolescent. *Pediatr Infect Dis J* **2020**, *39*, e314–e315, doi:10.1097/INF.0000000000002853.
37. Valente-Acosta, B.; Moreno-Sanchez, F.; Fueyo-Rodriguez, O.; Palomar-Lever, A. Rhabdomyolysis as an Initial Presentation in a Patient Diagnosed with COVID-19. *BMJ Case Rep* **2020**, *13*, e236719, doi:10.1136/bcr-2020-236719.
38. Wongkittichote, P.; Watson, J.R.; Leonard, J.M.; Toolan, E.R.; Dickson, P.I.; Grange, D.K. Fatal COVID-19 Infection in a Patient with Long-chain 3-hydroxyacyl-CoA Dehydrogenase Deficiency: A Case Report. *JIMD Rep* **2020**, *56*, 40–45, doi:10.1002/jmd2.12165.
39. Zhang, Q.; Shan, K.S.; Minalyan, A.; O'Sullivan, C.; Nace, T. A Rare Presentation of Coronavirus Disease 2019 (COVID-19) Induced Viral Myositis With Subsequent Rhabdomyolysis. *Cureus* **2020**, *12*, e8074, doi:10.7759/cureus.8074.
40. Zombori, L.; Bacon, M.; Wood, H.; Chatterjee, F.; Venkateswaran, R.; Lampariello, S.; Yoong, M. Severe Cortical Damage Associated with COVID-19 Case Report. *Seizure* **2021**, *84*, 66–68, doi:10.1016/j.seizure.2020.11.014.
41. Arai, R.; Murata, N.; Yamada, A.; Migita, S.; Koyama, Y.; Morikawa, T.; Ihara, S.; Akutsu, N.; Kuwana, T.; Fukamachi, D.; et al. Multimodal Findings of COVID-19-Related Rhabdomyolysis Complicated With Pericarditis Mimicking Fulminant Myocarditis. *Circ Rep 3*, 419–420, doi:10.1253/circrep.CR-21-0055.
42. Armanpoor, P.; Armanpoor, P.; Armanpoor, P.; Armanpoor, P. Pediatric COVID-19 Associated Rhabdomyolysis. *The Journal of Pediatric Research* **2021**, doi:10.4274/jpr.galenos.2021.27790.
43. Bach, M.; Lim, P.P.; Azok, J.; Ruda Wessell, K.; Desai, A.P.; Dirajlal-Fargo, S. Anaphylaxis and Rhabdomyolysis: A Presentation of a Pediatric Patient With COVID-19. *Clin Pediatr (Phila)* **2021**, *60*, 202–204, doi:10.1177/0009922821999470.
44. Bibbo, C. Reconstruction of COVID-19-Related Compartment Syndrome With Massive Soft Tissue Necrosis. *Wounds* **2021**, *33*, 99–105.
45. Byler, J.; Harrison, R.; Fell, L.L. Rhabdomyolysis Following Recovery from Severe COVID-19: A Case Report. *Am J Case Rep* **2021**, *22*, e931616-1-e931616-5, doi:10.12659/AJCR.931616.
46. Cassim, F.; Soni, A.J.; Murphy, S. Severe Acute Inflammatory Myositis and Rhabdomyolysis in Paediatric SARS-CoV-2-Associated MIS-C (Multisystem Inflammatory Syndrome in Children). *BMJ Case Rep* **2021**, *14*, e243112, doi:10.1136/bcr-2021-243112.
47. Chetram, V.K.; Ahmad, A.I.; Farid, S.; Sood, T. Acute Kidney Injury Secondary to Rhabdomyolysis and COVID-19: A Case Report and Literature Review. *Case Rep Nephrol* **2021**, *2021*, 5528461, doi:10.1155/2021/5528461.

48. Chiba, N.; Matsuzaki, M.; Mawatari, T.; Mizuochi, M.; Sakurai, A.; Kinoshita, K. Beneficial Effects of Dantrolene in the Treatment of Rhabdomyolysis as a Potential Late Complication Associated with COVID-19: A Case Report. *European Journal of Medical Research* **2021**, *26*, 18, doi:10.1186/s40001-021-00489-8.
49. Desai, S.; Quraishi, J.; Citrin, D. Prolonged Self-Resolving Neutropenia Following Asymptomatic COVID-19 Infection. *Cureus* **2021**, *13*, e16451, doi:10.7759/cureus.16451.
50. Egoryan, G.; Chaudry, S.; Yadav, K.; Dong, T.; Ozcekirdek, E.; Ozen, E.; Rodriguez-Nava, G. Dark Urine as the Initial Manifestation of COVID-19: A Case Report. *J Med Case Rep* **2021**, *15*, 576, doi:10.1186/s13256-021-03173-x.
51. Fabi, M.; Guida, F.; Pierantoni, L.; Greco, L.; di Luca, D.; Lanari, M. Severe Rhabdomyolysis in a Child With Multisystem Inflammatory Syndrome: An Autoimmune Mechanism? *Pediatr Neurol* **2021**, *124*, 11–12, doi:10.1016/j.pediatrneurol.2021.07.015.
52. Fadel, C.; Phan, N.; Kaur, A. Liberal Intravenous Fluid Administration in a Rare Case of Severe Rhabdomyolysis Secondary to SARS-CoV-2. *Cureus* **2021**, *13*, e17234, doi:10.7759/cureus.17234.
53. Fadiran, O. An Atypical Case of COVID-19 Induced Pancytopenia, Rhabdomyolysis and Myocarditis. *Cureus* **2021**, *13*, e12455, doi:10.7759/cureus.12455.
54. Farouji, A.; Hellou, R.; Peretz, A. Asymptomatic Rhabdomyolysis in a Young Adult With COVID-19. *Cureus* **2021**, *13*, e18039, doi:10.7759/cureus.18039.
55. Flato, U.A.P.; Ferreira, K.V.; Biteli, P.; Reina, D.O.B.R.; Reina, F.T.R.; Vernaschi, F.T.; Souza, G.A. de; Campanari, G.S. dos S.; Matias, J.N.; Lima, V.M.; et al. Rabdomyolysis as a Serious Complication of COVID-19. *AIMSBOA* **2021**, *8*, 165–172, doi:10.3934/bioeng.2021015.
56. Fujita, K.; Kanai, O.; Nanba, K.; Esaka, N.; Hata, H.; Seta, K.; Odagaki, T. Acute Rhabdomyolysis in a Young Woman with Moderate COVID-19. *IDCases* **2021**, *25*, e01212, doi:10.1016/j.idcr.2021.e01212.
57. Gilpin, S.; Byers, M.; Byrd, A.; Cull, J.; Peterson, D.; Thomas, B.; Jacobson, P. Rhabdomyolysis as the Initial Presentation of SARS-CoV-2 in an Adolescent. *Pediatrics* **2021**, *147*, e2020019273, doi:10.1542/peds.2020-019273.
58. Heidarpour, M.; Vakhshoori, M.; Haghighatpanah, M.A.; Ashrafi, L.; Khorvash, F.; Iraj, B. Rhabdomyolysis plus Hypocalcemia and Diabetic Ketoacidosis as Concurrent Rare COVID-19 Manifestations. *Case Rep Med* **2021**, *2021*, 6625086, doi:10.1155/2021/6625086.
59. Hookey, G.; Ahmad, Q.; McCune, T.; Kowalewska, J.; Amaker, B.; Inayat, N. Diagnostic Role of Technitium-99m Bone Scan in Severe COVID-19-Associated Myositis. *Radiol Case Rep* **2021**, *16*, 2123–2128, doi:10.1016/j.radcr.2021.05.019.
60. Lamzouri, O.; Bouchlarhem, A.; Haddar, L.; Elaidouni, G.; Es-saad, O.; Bkiyar, H.; Housni, B. SARS-CoV-2 Infection Presenting as Rhabdomyolysis: Case Report and Review. *J Int Med Res* **2021**, *49*, 03000605211061035, doi:10.1177/03000605211061035.
61. Li, A.P.Z.; Thomas, S.; Gokmen, R.; Kariyawasam, D. Rhabdomyolysis and Severe Biphasic Disturbance of Calcium Homeostasis Secondary to COVID-19 Infection. *BMJ Case Rep* **2021**, *14*, e239611, doi:10.1136/bcr-2020-239611.
62. Mahmood, U.A.; Mermis, J.D.; Khan, N.-M.; El Atrouni, W. Rhabdomyolysis With COVID-19. *Infect Dis Clin Pract (Baltim Md)* **2021**, *29*, e129–e130, doi:10.1097/IPC.0000000000000968.
63. Mughal, M.S.; Kaur, I.P.; Alhashemi, R.; Rehman, R.; Du, D. Acute Viral Myositis Complicated by Rhabdomyolysis: A Sole Manifestation of COVID-19 Infection. *J Community Hosp Intern Med Perspect* **2021**, *11*, 289–291, doi:10.1080/20009666.2021.1878601.

64. Patel, V.; Alzghoul, B.; Kalra, S.S. COVID-19 Infection and Severe Rhabdomyolysis. *Proc (Bayl Univ Med Cent)* **2021**, *34*, 478–480, doi:10.1080/08998280.2021.1897341.
65. Pérez, J.; Sánchez, S.; Sepúlveda, R.A.; Vera, M.; Mery, P.; Garayar, B.; Jalil, R. [Acute kidney injury caused by rhabdomyolysis as a SARS-CoV-2 infection manifestation. Report of one case]. *Rev Med Chil* **2021**, *149*, 641–647, doi:10.4067/s0034-98872021000300641.
66. Riccardi, J.; Fredericks, C.J.; Callcut, R.A. Trauma and COVID-Induced Severe Rhabdomyolysis. *Am Surg* **2022**, *88*, 1003–1005, doi:10.1177/00031348211063569.
67. Toro, L.; Zamorano, P.; Frías, A.; Parra-Lucares, A.; Silva, M.F.; Almeida, P.; Bozán, M.F.; Sanhueza, M.E.; Torres, R. [Rhabdomyolysis as the presentation form of COVID-19 infection. Report of one case]. *Rev Med Chil* **2021**, *149*, 796–802, doi:10.4067/s0034-98872021000500796.
68. Vasquez-Garagatti, R.; Díaz-Pardavé, C.; Beas, R.; Bieber, J.D.; Gnoni, M.; Granda-Irribarren, N. [Late rhabdomyolysis secondary to SARS-CoV-2 infection]. *Medicina (B Aires)* **2021**, *81*, 656–658.
69. Veyseh, M.; Koyoda, S.; Ayesha, B. COVID-19 IgG-Related Autoimmune Inflammatory Necrotizing Myositis. *BMJ Case Rep* **2021**, *14*, e239457, doi:10.1136/bcr-2020-239457.
70. Wu, H.-Y.; Pan, C.-T.; Cheng, C.-F.; Lin, C.-Y.; Chang, S.-N.; Chen, Y.-C.; Wang, C.-Y.; Chen, Y.-F.; Chen, C.-Y.; Ma, M.H.-M.; et al. Combined Intravenous Immunoglobulin and Baricitinib Treatment for Severe COVID-19 with Rhabdomyolysis: A Case Report. *J Formos Med Assoc* **2021**, *120*, 1777–1781, doi:10.1016/j.jfma.2021.03.014.
71. Abdelmottaleb, W.; Salmon, J.T.; Quintanilla Rodriguez, B.S.; Portillo, I.; Mushiyev, S. COVID-19 Myopericarditis With Pericardial Effusion Complicated With Cardiac Tamponade and Rhabdomyolysis. *Cureus* **2022**, *14*, e27291, doi:10.7759/cureus.27291.
72. Banamah, T.A.; Bogari, A.A.; Neyazi, A.; Kotbi, E.; Almaghraby, H.; Atwah, F. Severe Rhabdomyolysis Complicated With Acute Kidney Injury Required Renal Replacement Therapy After Pfizer COVID-19 Vaccine. *Cureus 14*, e25199, doi:10.7759/cureus.25199.
73. Bawor, M.; Sairam, S.; Rozewicz, R.; Viegas, S.; Comninos, A.N.; Abbara, A. Rhabdomyolysis after COVID-19 Infection: A Case Report and Review of the Literature. *Viruses* **2022**, *14*, 2255, doi:10.3390/v14102255.
74. Donati, G.; Abenavoli, C.; Vischini, G.; Cenacchi, G.; Costa, R.; Pasquinelli, G.; Ferracin, M.; Laprovitera, N.; Comai, G.; Monti, G.; et al. Sickle Cell Trait and SARS-CoV-2-Induced Rhabdomyolysis: A Case Report. *Am J Case Rep* **2022**, *23*, e934220, doi:10.12659/AJCR.934220.
75. Freund, O.; Eviatar, T.; Bornstein, G. Concurrent Myopathy and Inflammatory Cardiac Disease in COVID-19 Patients: A Case Series and Literature Review. *Rheumatol Int* **2022**, *42*, 905–912, doi:10.1007/s00296-022-05106-3.
76. Jahnke, V.S.; Poloni, J.A.T.; Neves, C.A.M.; Peter, C.; Thompson, C.E.; Rotta, L.N. Acute Kidney Injury Associated with Rhabdomyolysis in a Patient with COVID-19. *J Bras Nefrol* **2022**, *44*, 443–446, doi:10.1590/2175-8239-JBN-2020-0170.
77. Kar, R.; Murga, A.G.; Teruya, T.H.; Patel, S.T. COVID-19 Associated Rhabdomyolysis Leading to Major Amputation in the Absence of Macrovascular Thrombosis. *Ann Vasc Surg Brief Rep Innov* **2022**, *2*, 100082, doi:10.1016/j.avsurg.2022.100082.
78. Kermond, R.; Cavazzoni, E.; Kilo, T.; Britton, P.N.; Durkan, A. COVID-19-Associated Rhabdomyolysis in a Paediatric Patient with Sickle Cell Trait. *J Paediatr Child Health* **2022**, *58*, 1664–1665, doi:10.1111/jpc.15867.
79. Kontou, M.; Kakleas, K.; Kimioni, V.; Georgiadi, D.; Spoulou, V.; Michos, A. Rhabdomyolysis and Coronavirus Disease-2019 in Children: A Case Report and Review of the Literature. *Pediatr Investig* **2022**, *6*, 135–139, doi:10.1002/ped4.12320.

80. Mariano, J.; MacLaren, G.A. A Case of Rhabdomyolysis in a Young, Morbidly Obese, Asthmatic Woman With COVID-19. *Cureus 14*, e28950, doi:10.7759/cureus.28950.
81. Merbouh, M.; El Aidouni, G.; Serbource, J.; Couprie, L.M.; Bernardoni, C.; Housni, B.; Monchi, M. Isolated Severe Rhabdomyolisis Revealing COVID-19: Case Report. *Oxf Med Case Reports* **2022**, *2022*, omac039, doi:10.1093/omcr/omac039.
82. Miranda, L.H.L.; de Lima, D.N.; Dourado, M.M.C. An Unusual Presentation of Rhabdomyolysis and Acute Kidney Injury after Physical Activity: A Case Report. *Case Rep Nephrol Dial* **2022**, *12*, 193–200, doi:10.1159/000527194.
83. Mittal, N.; Del Rio-Pertuz, G.; Abohelwa, M. COVID-19 Causing Rhabdomyolysis Requiring Hemodialysis in a Young Adult. *Proc (Bayl Univ Med Cent)* **2022**, *35*, 510–511, doi:10.1080/08998280.2022.2051787.
84. Rambabova-Bushljetik, I.; Dzekova-Vidimliski, P.; Karanfilovski, V.; Sterjova-Markovska, Z.; Cana, F.; Bushljetikj, O.; Balkanov, S.; Kapedanovska-Nestorovska, A.; Dimovski, A. Rhabdomyolysis Associated with Recent SARS-COV-2 Infection in a Patient with Carnitine Palmitoyltransferase II Deficiency. *Pril (Makedon Akad Nauk Umet Odd Med Nauki)* **2022**, *43*, 61–66, doi:10.2478/prilozi-2022-0037.
85. Takada, K.; Sada, Y.; Samura, M.; Matsuura, M.; Hirose, N.; Kurata, T.; Nagumo, F.; Ishii, J.; Koshioka, S.; Uchida, M.; et al. A Case of Exacerbation of Haloperidol-Induced Rhabdomyolysis Following the Onset of COVID-19. *Am J Case Rep* **2022**, *23*, e936589, doi:10.12659/AJCR.936589.
86. Tuan, J.J.; Ogbuagu, O.; Kumar, D.; Altice, F.; Fikrig, M. Acute Kidney Injury Due to Myoglobin Cast Nephropathy in the Setting of Coronavirus Disease 2019-Mediated Rhabdomyolysis: A Case Report. *J Med Case Rep* **2022**, *16*, 491, doi:10.1186/s13256-022-03721-z.
87. Wu, P.-S.; Wong, S.-B.; Cheng, C.-F.; Yu, C.-H. Rhabdomyolysis in Pediatric Patients with SARS-CoV-2 Infection. *Children (Basel)* **2022**, *9*, 1441, doi:10.3390/children9101441.
88. Yu, R.; Chen, C.-R.; Evans, D.; Qing, X.; Gotesman, M.; Chandramohan, G.; Kallay, T.; Lin, H.J.; Pedigo, T.P. Glucose-6-Phosphate Dehydrogenase Deficiency Presenting with Rhabdomyolysis in a Patient with Coronavirus Disease 2019 Pneumonia: A Case Report. *J Med Case Rep* **2022**, *16*, 106, doi:10.1186/s13256-022-03322-w.
89. Geeting, D.; Alibrahim, O.; Patel, M.; Kumar, R.; Mallory, P. COVID-19 and Severe Rhabdomyolysis Causing Acute Kidney Injury and Life-Threatening Hyperkalemia in a Pediatric Patient: A Case Report. *SN Compr Clin Med* **2023**, *5*, 121, doi:10.1007/s42399-023-01453-9.
90. Hassan, M.A.; Batta, Y.; Smith, T.; Afzal, M.A. Non-ST Segment Elevation Myocardial Infarction (NSTEMI) in the Setting of Severe Rhabdomyolysis and COVID-19 Infection: A Case Report. *Cureus* **2023**, *15*, e40554, doi:10.7759/cureus.40554.
91. Khalayli, N.; Ibrahim, R.; Ibrahim, R.; Kudsi, M. Fatal Rhabdomyolysis Caused by COVID-19 Infection: A Case Report. *Ann Med Surg (Lond)* **2023**, *85*, 3683–3685, doi:10.1097/MS9.0000000000000881.
92. Lu, W.; Li, X.; You, W.; Gong, R. Rhabdomyolysis in a Patient with End-Stage Renal Disease and SARS-CoV-2 Infection: A Case Report. *Medicine (Baltimore)* **2023**, *102*, e36360, doi:10.1097/MD.0000000000036360.
93. Matsuki, Y.; Sugihara, T.; Kihara, T.; Kawakami, T.; Kitaura, T.; Takata, T.; Nagahara, T.; Fujita, K.; Hirai, M.; Kato, M.; et al. COVID-19-Triggered Acute Liver Failure and Rhabdomyolysis: A Case Report and Review of the Literature. *Viruses* **2023**, *15*, 1445, doi:10.3390/v15071445.
94. Sotirios, K.; Vasiliki, N.; Stamatoula, T.; Alexia, A.; Nikolaos, M.; Sylvia, R. A Case of Rhabdomyolysis and Weaning Failure in a Patient With Severe SARS CoV-2 Infection. *J Acute Med* **2023**, *13*, 75–78, doi:10.6705/j.jacme.202306_13(2).0004.
95. Dye, M.; Lantz, R. Profound Rhabdomyolysis and Viral Myositis Due to SARS-CoV-2: A Case Report. *Cureus 16*, e61172, doi:10.7759/cureus.61172.

96. Nayak M, V.; Bipasha, F.; Neelakantappa, K. Asymptomatic COVID-19 Infection-Induced Rhabdomyolysis in the Backdrop of Statin-Cyclosporine Drug Interaction. *Cureus* **2024**, *16*, e68127, doi:10.7759/cureus.68127.
97. Okubo, T.; Kouzu, H.; Kamada, A.; Endo, K.; Kawaharata, W.; Ohwada, W.; Suda, K.; Nagano, N.; Sakurai, A.; Koyama, M.; et al. Slowly Progressive Rhabdomyolysis Post COVID-19: Insights for Acute Kidney Injury Prediction With Discordant Creatine Kinase and Myoglobin Elevations. *Cureus* **2024**, *16*, e68145, doi:10.7759/cureus.68145.
98. Reddy, S.A.; Rivera Vargas, N.; Varshney, A.; Karasik, O. A Case of COVID-19-Triggered Polymyositis Leading to Rhabdomyolysis. *Cureus* **2024**, *16*, e64267, doi:10.7759/cureus.64267.
99. Sripathy, M.; Mishra, N.; Patil, A.; Siddini, V. Rhabdomyolysis in a Case of Mild Coronavirus Disease 2019 and β-Thalassemia Minor. *J Assoc Physicians India* **2024**, *72*, 97–99, doi:10.59556/japi.72.0612.
100. Yuan, S.; Huang, Y.; Xie, P.; Li, P. A Case of Severe Rhabdomyolysis, Acute Myocardial Damage and Multi-Organ Dysfunction Syndrome in a Patient with Novel Coronavirus Pneumonia. *Open Access Emerg Med* **2024**, *16*, 19–28, doi:10.2147/OAEM.S446994.
