# Supplementary material for: Rhabdomyolysis Reported in Association with SARS-CoV-2 Infection and COVID-19 Vaccination: A Systematic Review
Source: Medicina (Kaunas). 2026 Sep 15;62(9):1769. doi: 10.3390/medicina62091769 (PMC13608938; doi:10.3390/medicina62091769)
Supplement: Supplementary file 1 [file medicina-62-01769-s001.zip › SM3. Table S2.pdf]

Table S2. Definition and ascertainment of rhabdomyolysis among included observational studies

First author/year	Definition of Rhabdomyolysis	Threshold type	CK threshold	Clinical symptoms/myoglobinuria	Timing of onset	Alternative causes
Albaba et al. 2021[20]	CK > 5 times the ULN	ULN (225 U/L)	> 5 x ULN	Not required	During hospitalization	NR
Ali et al. 2021[21]	CK > 1 000 U/L	Absolute value	> 1 000 U/L	Not required	During hospitalization	NR
Geng et al. 2021[22]	CK > 5 times the ULN<br>CK-MB < 5% of total CK	ULN (170 U/L)	> 5 x ULN	Not required	During hospitalization	NR
Haroun et al. 2021[23]	CK > 1 000 U/L	Absolute value	> 1 000 U/L	Not required	During hospitalization	NR
Martinot et al. 2021[24]	CK ≥ 3 ULN	ULN (not specified)	> 3 x ULN	Not required	During hospitalization	NR
Mohamed et al. 2021[25]	CK ≥ 5 000 U/L<br>≥2+ heme by dipstick	Absolute value	≥ 5 000 U/L	Not required	During hospitalization	NR
Mokhtari et al. 2021[26]	CK > 1 000 U/L	Absolute value	> 1 000 U/L	Not required	Admission to ICU	NR
Zamoner et al. 2021[27]	NR	NR	NR	NR	During hospitalization	NR
Khatib et al. 2022[28]	NR	NR	NR	Not required	Stay in ICU	NR
Nashwan et al. 2023[29]	CK > 5 times the ULN<br>or CK: 1 500 – 100 000 U/L<br>Myoglobin > 8' ng/mL	ULN<br>Absolute value	> 5 x ULN<br>> 1 000 U/L	Not required	Stay in ICU	NR
Hashemi et al. 2024[30]	CK ≥ 1 000 U/L	Absolute value	≥ 1 000 U/L	Not required	During hospitalization	NR
Murt and Altiparmak 2024	CK > 5 times the ULN	ULN (not specified)	> 5 x ULN	Not required	During hospitalization	NR
Samardzic et al. 2024[31]	CK ≥ 1 000 U/L<br>Myoglobinuria	Absolute value	≥ 1 000 U/L	Not required	During hospitalization	NR

CK: creatine kinase; CK-MB: creatine kinase-myocardial band; ICU: intensive care unit; NR: not reported; ULN: upper limit of normal.
