# Supplementary material for: Rhabdomyolysis Reported in Association with SARS-CoV-2 Infection and COVID-19 Vaccination: A Systematic Review
Source: Medicina (Kaunas). 2026 Sep 15;62(9):1769. doi: 10.3390/medicina62091769 (PMC13608938; doi:10.3390/medicina62091769)
Supplement: Supplementary file 1 [file medicina-62-01769-s001.zip › SM4. Table S3.pdf]

Table S3. New-onset Rhabdomyolysis induced by COVID-19 vaccines.

First author name/year of publication	Country	Design	Age/Sex	Antecedentes-comorbilidades	Type of vaccine	Dose	Time to symptom onset	Clinical Manifestations	Physical Exam	Lab tests	Imaging	Urinalysis (myoglobinuria)	Diagnosis	Complication	Treatment	Development
Ajmera 2021[1]	USA	Case report	85 F	Rheumatoid arthritis Hyperlipidemia Asthma CVA	Moderna (mRNA)	2nd	2 days	Generalized weakness Muscle cramps Loss of appetite Dark urine	Confused Edema over the bilateral upper and lower extremities	CK: >14000 U/L BUN: 73 mg/dL AST: 1422 U/L ALT: 600 U/L	None	Positive	Rhabdomyolysis	Hypoxia Renal failure Anasarca Cardiac arrest	IV fluids Glucocorticoids Ventilator support	Death
Elias et al. 2021[2]	Portugal	Case report	81 M	IHD CVD Idiopathic pulmonary fibrosis	Pfizer BioNTech 162b2 (mRNA)	1st	3 days	Generalized weakness	Normal	CK: 7799 U/L Myoglobin: 12000 ng/mL	None	NR	Rhabdomyolysis	None	IV fluids	Favorable
Gelbenegger et al. 2021[3]	Austria	Case report	19 M	None	Johnson & Johnson (viral vector)	1st	2 days	Myalgia Muscle weakness	Normal	CK: 44180 U/L Creatinine:	NR	Positive	Rhabdomyolysis	None	IV fluids Acetaminophen	Favorable

								Dark urine		1.06 mg/dL<br>AST: 340 U/L						
Hakroush and Tampe 2021[4]	Germany	Case report	79 F	HBP<br>Degenerative disc disease	Pfizer BioNTech 162b2 (mRNA)	2nd	14 days	Weakness muscle<br>Myalgia	Normal	CK: 14243 U/L<br>Creatinine: 6.5 mg/dL	None	Negative	Rhabdomyolysis	ANCA-Associated Vasculitis<br>Pauci-Immune Crescentic Glomerulonephritis	IV fluids<br>Steroids<br>Cyclophosphamide	Favorable
Mack et al. 2021[5]	USA	Case report	80 M	DM2<br>COVID-19 infection	Moderna (mRNA)	2nd	2 days	Generalized body aches<br>Difficulty with ambulation	Normal	CK: 6546 U/L<br>AST: 112 U/L	None	Negative	Rhabdomyolysis	None	IV fluids	Favorable
Nassar et al. 2021[6]	USA	Case report	21 M	Asthma	Pfizer BioNTech 162b2 (mRNA)	1st	1 day	Backache<br>Swelling<br>Dark urine	Positive for tenderness to the paraspinal lumbar area upon palpation	CK: 22000 U/L<br>AST: 675 U/L	None	NR	Rhabdomyolysis	None	Morphine	Favorable

Tan et al. 2021[7]	UK	Case report	27 M	CPT II deficiency	Oxford/AstraZeneca (viral vector)	1st	5 hours	Generalized illness<br>Myalgia<br>Vomiting<br>Fever<br>SOB	Muscle weakness	CK: 102560 U/L	None	NR	Rhabdomyolysis	None	IV dextrose<br>Paracetamol	Favorable
Al-Rasbi et al. 2022[8]	Oman	Case report	37 M	None	Pfizer BioNTech 162b2 (mRNA)	1st	12 days	Back pain<br>Swelling<br>Paresthesia<br>SOB	Reduction in sensation to fine touch<br>Tenderness at paraspinal muscles	CK: 93046 U/L<br>CRP: 11 mg/L	MRI: diffuse subcutaneous edema<br>US: tissue edema, abnormal heterogeneous echogenicity	NR	Rhabdomyolysis	AKI	IV normal saline<br>Sodium bicarbonate<br>Furosemide<br>Tramadol<br>Paracetamol<br>IV immunoglobulin<br>Methylprednisolone	Favorable
Cirillo et al. 2022[9]	Italy	Case report	68 M	None	Oxford/AstraZeneca (viral vector)	1st	9 days	Dyspnea<br>Abdominal pain<br>Myalgia<br>Difficulty with ambulation	Weakness in the four limbs	CK: 376 U/L<br>Creatinine: 3.7 mg/dL<br>AST: 7815 U/L	Eco-Doppler: deep vein thrombosis	NR	Rhabdomyolysis	Multi organ failure	Steroids<br>Anakinra<br>Eculizumab	Death

Huang et al. 2022[10]	Taiwan	Case report	44 M	None	Oxford/AstraZeneca (viral vector)	2nd	14 days	Weakness<br>Dark urine	Bilateral forearm and gastrocnemius muscles were tense	CK: 151058 U/L<br>Creatinine: > 4.5 mg/dL	CT: retroperitoneal fluid collection and swelling and edematous change of his psoas muscles	NR	Rhabdomyolysis	Myositis	Fasciotomy<br>Methylprednisolone<br>Cyclophosphamide	Death
Kamura et al. 2022[11]	Japan	Case report	57 M	None	Moderna (mRNA)	1st	14 days	Leg pain	Livedo reticularis on the skin of the extremities	CK: 12096 U/L	MRI: on T2 weighted d-imaging demonstrates increased signal intensity in the thigh muscles	NR	Rhabdomyolysis	Severe kidney failure<br>Massive bleeding<br>Multiple thrombosis of the small arteries	Eculizumab<br>Methylprednisolone<br>Argatroban<br>Heparin<br>IVIG	Death
Kalekar et al. 2022[12]	India	Case report	31 F	None	Oxford/AstraZeneca (viral vector)	1st	10 days	Lower limb progressive swelling and pain	NR	CK: 15000 U/L<br>LDH: 350 U/L	US: extensive subcutaneous edema<br>MRI: abnormal signal	NR	Rhabdomyolysis	NR	Prednisolone<br>IV fluids	Favorable

											changes in T2WI and STIR sequences					
Pucchio et al. 2022[13]	Canada	Case report	16 M	None	Pfizer BioNTech 162b2 (mRNA)	1st	1 day	Dark urine<br>Myalgia	Limited range of motion of his upper extremities	CK: 147600 U/L	None	Positive	Rhabdomyolysis	None	IV hydration	Favorable
Ruijters et al. 2022[14]	The Netherlands	Case report	80 M	Asthma<br>HBP<br>Myocardial infarction<br>COPD	Pfizer BioNTech 162b2 (mRNA)	2nd	1 day	Myalgia<br>Fatigue<br>Muscle weakness<br>Malaise<br>Nausea	Tender muscles during palpation and generalized muscle weakness	CK: 280600 u/L<br>CRP: 14 mg/dL	None	NR	Rhabdomyolysis	None	IV immunoglobulin<br>IV fluids	Favorable
Sutcu et al. 2022[15]	Turkey	Case report	16 M	None	Pfizer BioNTech 162b2 (mRNA)	2nd	2 days	Generalized weakness<br>Muscle pain	Slightly decreased muscle strength	CK: 71339 U/L<br>CK-MB: 58 U/L	US: mild hepatic steatosis	NR	Rhabdomyolysis	None	IV hydration	Favorable
Unger et al. 2022[16]	USA	Case report	69 F	DM2<br>Obesity<br>HBP	Pfizer BioNTech 162b2 (mRNA)	2nd	1 day	Dark urine<br>Nocturia	NR	CK: 8394 U/L	None	NR	Rhabdomyolysis	AKI	Normal saline<br>Lactated ringers	Favorable

				Hyperlipidemia<br>Asthma<br>Pedal edema<br>CAD<br>Osteoarthritis<br>Chronic pain				Groin pain during urination		Creatinine: 2.7 mg/dL<br>AST: 235 U/L<br>ALT: 211 U/L						
Sheka et al. 2023[17]	Switzerland	Case report	20 M	None	Moderna (mRNA)	1st	2 days	Muscle pain<br>Swelling	Mild swelling of the elbows and forearms	CK: 145000 U/L<br>ALT: 687 U/L<br>AST: 1930 U/L<br>LDH: 1850 U/L	US: hyperechogenic patches within the biceps brachii and wrist flexor muscles	Positive	Rhabdomyolysis	None	Hyper-hydration with IV normal saline	Favorable
Ghiletchi et al. 2024[18]	Portugal	Case report	52 M	DM<br>HBP<br>Asthma<br>Dyslipidemia	Pfizer BioNTech 162b2 (mRNA)	5th	2 days	Severe pain<br>Muscle weakness	Diminished muscle strength with inability to walk	CK: 400000 U/L<br>CRP: 92 mg/L<br>AST: 1806 U/L<br>ALT: 380 U/L	None	Positive	Rhabdomyolysis	None	IV fluids<br>Oral hydration<br>Rosuvastatin	Favorable

Hazari et al. 2025[19]	USA	Case report	53 F	DM Dyslipidemia	Pfizer BioNTech 162b2 (mRNA)	2nd	45 days	Extremity weakness Muscle pain	Bilateral proximal weakness in the upper and lower extremities	CK: 7800 U/L ALT: 384 U/L AST: 241 U/L	MRI: mild degenerative changes in the discs	NR	Rhabdomyolysis	None	IV hydration Prednisone IVIG	Favorable
Ueda et al. 2025[20]	Japan	Case report	60 M	Dyslipidemia Schizophrenia	mRNA vaccine	4th	2 days	Muscle pain Anuria	Bilateral lower leg grasping pain	CK: 160000 U/L Creatinine: 6.6 mg/dL	CT: normal	Positive	Rhabdomyolysis	AKI	IV fluid therapy RRT	Favorable

AKI: acute kidney injury; ALT: alanine aminotransferase; AST: aspartate aminotransferase; BUN: blood urea nitrogen; CAD: coronary artery disease; CK: creatinine kinase; CK-MB: creatine kinase-myoglobin binding; COPD: chronic obstructive pulmonary disease; CPT II: Carnitine palmitoyltransferase II; CVA: cerebrovascular accident; CVD: cerebrovascular disease; DM: diabetes mellitus; DM2: type II diabetes mellitus; ECG: electrocardiogram; F: female; HBP: high blood pressure; hs-CRP: high sensitive C-reactive protein; IHD: ischemic heart disease; IV: intravenous; IVIG: intravenous immunoglobulin; LDH: Lactate dehydrogenase; M: male; MRI: magnetic resonance imaging; NR: not reported; US: ultrasound; RRT: renal replacement therapy; SOB: shortness of breath.

REFERENCES

1. Ajmera, K.M. Fatal Case of Rhabdomyolysis Post-COVID-19 Vaccine. *Infect Drug Resist* **2021**, *14*, 3929–3935, doi:10.2147/IDR.S331362.
2. Elias, C.; Cardoso, P.; Gonçalves, D.; Vaz, I.; Cardoso, L. Rhabdomyolysis Following Administration of Comirnaty®. *Eur J Case Rep Intern Med* **2021**, *8*, 002796, doi:10.12890/2021_002796.
3. Gelbenegger, G.; Cacioppo, F.; Firbas, C.; Jilma, B. Rhabdomyolysis Following Ad26.COV2.S COVID-19 Vaccination. *Vaccines (Basel)* **2021**, *9*, 956, doi:10.3390/vaccines9090956.
4. Hakroush, S.; Tampe, B. Case Report: ANCA-Associated Vasculitis Presenting With Rhabdomyolysis and Pauci-Immune Crescentic Glomerulonephritis After Pfizer-BioNTech COVID-19 mRNA Vaccination. *Front Immunol* **2021**, *12*, 762006, doi:10.3389/fimmu.2021.762006.
5. Mack, M.; Nichols, L.; Guerrero, D.M. Rhabdomyolysis Secondary to COVID-19 Vaccination. *Cureus* **2021**, *13*, e15004, doi:10.7759/cureus.15004.
6. Nassar, M.; Chung, H.; Dhayaparan, Y.; Nyein, A.; Acevedo, B.J.; Chicos, C.; Zheng, D.; Barras, M.; Mohamed, M.; Alfishawy, M.; et al. COVID-19 Vaccine Induced Rhabdomyolysis: Case Report with Literature Review. *Diabetes Metab Syndr* **2021**, *15*, 102170, doi:10.1016/j.dsx.2021.06.007.
7. Tan, A.; Stepien, K.M.; Narayana, S.T.K. Carnitine Palmitoyltransferase II Deficiency and Post-COVID Vaccination Rhabdomyolysis. *QJM* **2021**, *114*, 596–597, doi:10.1093/qjmed/hcab077.
8. Al-Rasbi, S.; Al-Maqbali, J.S.; Al-Farsi, R.; Al Shukaili, M.A.; Al-Riyami, M.H.; Al Falahi, Z.; Al Farhan, H.; Al Alawi, A.M. Myocarditis, Pulmonary Hemorrhage, and Extensive Myositis with Rhabdomyolysis 12 Days After First Dose of Pfizer-BioNTech BNT162b2 mRNA COVID-19 Vaccine: A Case Report. *Am J Case Rep* **2022**, *23*, e934399, doi:10.12659/AJCR.934399.
9. Cirillo, E.; Esposito, C.; Giardino, G.; Azan, G.; Fecarotta, S.; Pittaluga, S.; Ruggiero, L.; Barretta, F.; Frisso, G.; Notarangelo, L.D.; et al. Case Report: Severe Rhabdomyolysis and Multiorgan Failure After ChAdOx1 nCoV-19 Vaccination. *Front Immunol* **2022**, *13*, 845496, doi:10.3389/fimmu.2022.845496.
10. Huang, S.-T.; Lee, T.-J.; Chen, K.-H.; Sun, H.-Y.; Chen, W.-T.; Hsieh, S.-C.; Cheng, A.; Chen, Y.-C. Fatal Myositis, Rhabdomyolysis and Compartment Syndrome after ChAdOx1 nCoV-19 Vaccination. *J Microbiol Immunol Infect* **2022**, *55*, 1131–1133, doi:10.1016/j.jmii.2022.04.003.
11. Kamura, Y.; Terao, T.; Akao, S.; Kono, Y.; Honma, K.; Matsue, K. Fatal Thrombotic Microangiopathy with Rhabdomyolysis as an Initial Symptom after the First Dose of mRNA–1273 Vaccine: A Case Report. *Int J Infect Dis* **2022**, *117*, 322–325, doi:10.1016/j.ijid.2022.02.031.
12. Kalekar, T.M.; Jaipuria, R.K.; Navani, R.S. MRI Findings in Case of Post-COVID-19 Vaccination Rhabdomyolysis: A Rare Postvaccination Adverse Effect. *Indian J Radiol Imaging* **2022**, *32*, 256–259, doi:10.1055/s-0042-1748534.
13. Pucchio, A.; Akiva, M.H.; Evangeliou, H.; Papenburg, J.; Salvadori, M.I. Severe Rhabdomyolysis Secondary to COVID-19 mRNA Vaccine in a Teenager. *Pediatr Nephrol* **2023**, *38*, 1979–1983, doi:10.1007/s00467-022-05808-7.
14. Ruijters, V.J.; van der Meulen, M.F.G.; van Es, M.A.; Smit, T.; Hoogendijk, J.E. Rhabdomyolysis after COVID-19 Comirnaty Vaccination: A Case Report. *Case Rep Neurol* **2022**, *14*, 429–432, doi:10.1159/000527599.
15. Sutcu, M.; Gul, D.; Atik, F.; Kara, M. Rhabdomyolysis after BNT162b2 mRNA Covid-19 Vaccine in an Adolescent Male. *Malawi Med J* **2022**, *34*, 154–156, doi:10.4314/mmj.v34i2.13.
16. Unger, K.; Ponte, C.D.; Anderson, D. A Possible Case of COVID-19 Booster Vaccine-Associated Rhabdomyolysis and Acute Kidney Injury. *J Pharm Technol* **2022**, *38*, 247–250, doi:10.1177/87551225221093944.
17. Sheka, M.; Coattrenec, Y.; Lorenzini, K.I.; Nendaz, M. A Severe Case of Rhabdomyolysis after Moderna mRNA Anti-COVID-19 Vaccine with a Literature Review. *Clin Case Rep* **2023**, *11*, e7184, doi:10.1002/ccr3.7184.
18. Ghiletchi, A.; Leão, R.; Coelho, C.; Ferreira, I.; Bernardino, R. Polymyositis and Severe Rhabdomyolysis in the Context of Influenza and COVID-19 Vaccination: A Case Report. *Cureus* **2024**, *16*, e66957, doi:10.7759/cureus.66957.

19. Hazari, A.; Chien, J.; Nunez, D. COVID-19 Vaccine-Induced Rhabdomyolysis in a 53-Year-Old Woman. *HCA Healthc J Med* **2025**, *6*, 259–262, doi:10.36518/2689-0216.1463.
20. Ueda, M.; Uchimura, K.; Ohkoshi, K.; Saegusa, N.; Osano, K.; Yoshida, S.; Konishi, M.; Ishii, T.; Takahashi, K.; Nakashima, A. A Severe Case of Rhabdomyolysis Requiring Renal Replacement Therapy Following COVID-19 mRNA Vaccination. *Intern Med* **2025**, *64*, 1547–1551, doi:10.2169/internalmedicine.4835-24.
