# Supplementary material for: Rhabdomyolysis Reported in Association with SARS-CoV-2 Infection and COVID-19 Vaccination: A Systematic Review
Source: Medicina (Kaunas). 2026 Sep 15;62(9):1769. doi: 10.3390/medicina62091769 (PMC13608938; doi:10.3390/medicina62091769)
Supplement: Supplementary file 1 [file medicina-62-01769-s001.zip › SM5. Table S4.pdf]

Table S4.1. JBI tool for assessing risk of bias and quality of case report studies.

Author/Question	Q1	Q2	Q3	Q4	Q5	Q6	Q7	Q8
**New-onset Rhabdomyolysis induced by SARS-CoV-2 infection**								
Alrubaye and Choudhary 2020	Yes	Yes	Yes	Yes	Yes	Yes	Yes	Yes
Anklesaria et al. 2020	Yes	Yes	Yes	No	Yes	No	Yes	Yes
Aria et al. 2020	Yes	Unclear	Yes	Unclear	Yes	Unclear	Unclear	Yes
Anwar and Al Lawati 2020	Yes	Unclear	Yes	No	Unclear	No	Yes	Yes
Case et al. 2020	Yes	Yes	Yes	Yes	Yes	Yes	Yes	Yes
Chan et al. 2020	No	No	Unclear	Unclear	Yes	Unclear	Unclear	Yes
Chedid et al. 2020	Yes	Yes	Yes	Yes	Yes	Yes	Yes	Yes
Chong and Saha 2020	Yes	Yes	Yes	Yes	Yes	Yes	Yes	Yes
Cunha et al. 2020	Yes	Yes	Yes	Yes	Yes	No	Yes	Yes
Foster et al. 2020	Yes	Yes	Yes	Yes	Yes	No	Yes	Yes
Fujimaru et al. 2020	Yes	Yes	Yes	Yes	Yes	Yes	Yes	Yes
Gefen et al. 2020	Yes	Yes	Yes	Yes	Yes	Yes	Yes	Yes
Husain et al. 2020	Yes	Yes	Yes	Yes	Yes	Unclear	Yes	Yes
Jin and Tong. 2020	Yes	Yes	Yes	Yes	Yes	Yes	Yes	Yes
Kajani et al. 2020	Yes	Yes	Yes	Yes	Yes	Yes	Yes	No
Kolkova et al. 2020	Yes	Yes	Yes	Yes	Yes	Yes	Yes	Yes
Legrand et al. 2020	Yes	Unclear	Yes	Yes	Unclear	No	Yes	Yes
Mah et al. 2020	Yes	Unclear	Yes	Yes	Unclear	No	Yes	Yes
Meegada et al. 2020	Yes	Yes	Yes	Yes	Yes	Yes	Yes	Yes
Mukherjee et al. 2020	Yes	Yes	Yes	Yes	Yes	Yes	Yes	Yes
Munoz-Martinez et al. 2020	Yes	Yes	Yes	Yes	Yes	Yes	Yes	Yes
Murillo et al. 2020	Yes	Yes	Yes	Yes	Yes	Yes	Yes	Yes
Rivas-García et al. 2020	Yes	Yes	Yes	Unclear	Yes	Yes	Not applicable	Yes
Rosato et al. 2020	Yes	Yes	Yes	Yes	Yes	Yes	Yes	Yes

Samies et al. 2020	Yes	Yes	Unclear	Yes	Yes	Yes	Yes	Yes
Sathappan et al. 2020	Yes	Yes	Yes	Yes	Yes	Yes	Yes	Yes
Shanbhag et al. 2020	Yes	Yes	Yes	Yes	Yes	No	Unclear	Unclear
Solís et al. 2020	Yes	Yes	Unclear	Yes	Yes	Yes	Not applicable	Yes
Suwanwongse and Shabarek 2020	Yes	Yes	Yes	Yes	Yes	No	No	No
Taxbro et al. 2020	Yes	Yes	Yes	Yes	Yes	Yes	Yes	Yes
Tram et al. 2020	Yes	Yes	Yes	Yes	Yes	Yes	Not applicable	Yes
Uysal et al. 2020	Yes	Yes	Unclear	Yes	Yes	Yes	Not applicable	Yes
Valente-Acosta et al. 2020	Yes	No	Yes	Unclear	Yes	Yes	Not applicable	Yes
Wongkittichote et al. 2020	Yes	Yes	Yes	Yes	Yes	Yes	Yes	Yes
Zhang et al. 2020	Yes	No	Yes	Unclear	Yes	Yes	Not applicable	Yes
Zombori et al. 2020	Yes	Yes	Yes	Yes	Yes	Yes	Yes	Not applicable
Arai et al. 2021	Yes	No	Yes	Yes	Unclear	Unclear	Not applicable	Unclear
Armanpoor et al. 2021	Yes	Yes	Yes	Yes	Yes	Yes	Yes	Yes
Bach et al. 2021	No	Yes	Yes	No	Yes	Yes	Yes	Yes
Bibbo 2021	No	Yes	Yes	No	Yes	Yes	Yes	Yes
Byler et al. 2021	Yes	Yes	Yes	Yes	Yes	No	Yes	Yes
Cassim et al. 2021	Yes	Unclear	Yes	Yes	Unclear	No	Yes	Yes
Chetram et al. 2021	Yes	Yes	Yes	Yes	Yes	Yes	Yes	Yes
Chiba et al. 2021	Yes	Yes	Yes	Yes	Yes	Yes	Unclear	Not applicable
Desai et al. 2021	Yes	Yes	Yes	Yes	Yes	Yes	Unclear	Not applicable
Egoryan et al. 2021	Yes	Yes	Yes	Yes	Yes	Yes	Unclear	Not applicable

Fabi et al. 2021	Yes	Yes	Yes	Yes	Yes	Yes	Yes	Not applicable
Fadel et al. 2021	Yes	Yes	Yes	Yes	Yes	Yes	Yes	Yes
Fadiran 2021	Yes	Unclear	Yes	Yes	Yes	Yes	Yes	Yes
Farouji et al. 2021	Yes	Yes	Unclear	Yes	Yes	Unclear	Yes	Yes
Flato et al. 2021	Yes	Yes	Yes	Yes	Yes	Yes	Yes	Not applicable
Fujita et al. 2021	Yes	Yes	Yes	Yes	Yes	Yes	Yes	Not applicable
Gilpin et al. 2021	Yes	Yes	Yes	Yes	Yes	No	Yes	Yes
Lamzouri et al. 2021	Yes	Yes	Yes	Yes	Yes	No	Yes	Yes
Li et al. 2021	Yes	Yes	Yes	Yes	Yes	Yes	Yes	Not applicable
Mahmood et al. 2021	Yes	Yes	Yes	Yes	Yes	Yes	Yes	Yes
Mughal et al. 2021	Yes	Yes	Yes	Yes	Yes	Yes	Yes	Not applicable
Patel et al. 2021	Yes	Yes	Yes	Yes	Yes	Yes	Unclear	Yes
Pérez et al. 2021	Yes	Yes	Yes	Yes	Yes	Yes	Yes	Unclear
Riccardi et al. 2021	Yes	Yes	Unclear	Yes	Yes	Yes	Not applicable	Yes
Toro et al. 2021	Yes	Yes	Unclear	Yes	Yes	Yes	Not applicable	Yes
Vasquez-Garagatti et al. 2021	Yes	Yes	Yes	Unclear	Yes	Yes	Yes	Yes
Veyseh et al. 2021	Yes	Yes	Yes	Yes	Yes	Yes	Yes	Not applicable
Wu et al. 2021	Yes	Yes	Unclear	Yes	Yes	Yes	Not applicable	Yes
Abdelmottaleb et al. 2022	Yes	Yes	Yes	Yes	Yes	Yes	Yes	Yes
Banamah et al. 2022	Yes	Yes	Yes	Yes	Yes	No	Yes	Yes
Bawor et al. 2022	Yes	Yes	Yes	Yes	Yes	Yes	Yes	Yes
Donati et al. 2022	Yes	Unclear	Yes	Yes	Unclear	No	Yes	Yes

Freund et al. 2022	Yes	Yes	Yes	Yes	Yes	Yes	Yes	Not applicable
Jahnke et al. 2022	Yes	Yes	Yes	Yes	Yes	Yes	Yes	Not applicable
Kar et al. 2022	Yes	Yes	Yes	Yes	Yes	Yes	Yes	Not applicable
Kermond et al. 2022	Yes	Yes	Unclear	Yes	Yes	Yes	Not applicable	Yes
Kontou et al. 2022	Yes	Yes	Yes	Yes	Yes	Yes	Yes	Yes
Mariano and MacLaren 2022	Yes	Yes	Yes	Yes	Yes	No	Yes	Yes
Merbouh et al. 2022	Yes	Yes	Yes	Yes	Yes	Yes	Yes	Yes
Miranda et al. 2022	Yes	Yes	Yes	Yes	Yes	Yes	Yes	Yes
Mittal et al. 2022	Yes	Yes	Yes	Yes	Yes	Yes	Yes	Yes
Rambabova-Bushljetik et al. 2022	Yes	Yes	Yes	Yes	Yes	Yes	Yes	Yes
Takada et al. 2022	Yes	Yes	Yes	Yes	Yes	Yes	Yes	Yes
Tuan et al. 2022	Yes	No	Yes	Unclear	Yes	Yes	Not applicable	Yes
Yu et al. 2022	Yes	No	Yes	Unclear	Yes	Yes	Not applicable	Yes
Geeting et al. 2023	Yes	Yes	Yes	Yes	Yes	Yes	Yes	Yes
Hassan et al. 2023	Yes	Unclear	Yes	Yes	Unclear	No	Yes	Yes
Khalayli et al. 2023	Yes	Yes	Yes	Yes	Yes	No	Yes	Yes
Lu et al. 2023	Yes	Yes	Yes	Yes	Yes	Unclear	Yes	Not applicable
Matsuki et al. 2023	Yes	Yes	Yes	Yes	Yes	Yes	Yes	Yes
Sotirios et al. 2023	Yes	No	Yes	Unclear	Yes	Yes	Not applicable	Yes
Dye and Lantz 2024	Yes	Yes	Yes	Yes	Yes	Yes	Yes	Yes
Nayak et al. 2024	Yes	Yes	Yes	Yes	Yes	Yes	Yes	Yes
Okubo et al. 2024	Yes	Yes	Yes	Yes	Yes	Yes	Yes	Yes
Reddy et al. 2024	Yes	Yes	Yes	Yes	Yes	Unclear	Yes	Yes
Sripathy et al. 2024	Yes	Yes	Unclear	Yes	Yes	Yes	Not applicable	Yes

Yuan et al. 2024	Yes	Yes	Unclear	Yes	Yes	Yes	Not applicable	Yes
**New-onset Rhabdomyolysis induced by COVID-19 vaccines**								
Ajmera 2021	Yes	Yes	Yes	Yes	Yes	Yes	Yes	Yes
Elias et al. 2021	Yes	Yes	Yes	Yes	Yes	Yes	Yes	Yes
Gelbenegger et al. 2021	Yes	Yes	Yes	Yes	Yes	Yes	Yes	Yes
Hakroush and Tampe 2021	Yes	Yes	Yes	Yes	Yes	Yes	Yes	Yes
Mack et al. 2021	Yes	Yes	Yes	Yes	Yes	Yes	Yes	Yes
Nassar et al. 2021	Yes	Yes	Yes	Yes	Yes	Yes	Yes	Yes
Tan et al. 2021	Yes	Yes	Yes	Yes	Yes	Yes	Yes	Yes
Al-Rasbi et al. 2022	Yes	Yes	Yes	Yes	Yes	Yes	Yes	Yes
Cirillo et al. 2022	Yes	Yes	Yes	Yes	Yes	Yes	Yes	Yes
Huang et al. 2022	Yes	Yes	Yes	Yes	Yes	Yes	Yes	Yes
Kamura et al. 2022	Yes	Yes	Yes	Yes	Yes	Yes	Yes	Yes
Kalekar et al. 2022	Yes	Yes	Yes	Yes	Yes	No	Yes	Yes
Pucchio et al. 2022	Yes	Yes	Yes	Yes	Yes	Yes	Yes	Yes
Ruijters et al. 2022	Yes	Yes	Yes	Yes	Yes	Yes	Yes	Yes
Sutcu et al. 2022	Yes	Yes	Yes	Yes	Yes	No	Yes	Yes
Unger et al. 2022	Yes	Yes	Yes	Yes	Yes	No	Yes	Yes
Sheka et al. 2023	Yes	Yes	Yes	Yes	Yes	Yes	Yes	Yes
Ghiletchi et al. 2024	Yes	Yes	Yes	Yes	Yes	Yes	Yes	Yes
Hazari et al. 2025	Yes	Yes	Yes	Yes	Yes	Yes	Yes	Yes
Ueda et al. 2025	Yes	Yes	Yes	Yes	Yes	Yes	Yes	Yes

Q1: Were patient's demographic characteristics clearly described? **Q2**: Was the patient's history clearly described and presented as a timeline? **Q3**: Was the current clinical condition of the patient on presentation clearly described? **Q4**: Were diagnostic tests or assessment methods and the results clearly described? **Q5**: Was the intervention(s) or treatment procedure(s) clearly described? **Q6**: Was the post-intervention clinical condition clearly described? **Q7**: Were adverse events (harms) or unanticipated events identified and described? **Q8**: Does the case report provide takeaway lessons?

Table S4.2. JBI tool for assessing risk of bias and quality of case series studies.

**Author/Question**	**Q1**	**Q2**	**Q3**	**Q4**	**Q5**	**Q6**	**Q7**	**Q8**	**Q9**	**Q10**
Buckholz et al. 2020	Yes	No	Yes	Unclear	Yes	Yes	Not applicable	Yes	Yes	Unclear
Khosla et al. 2020	Unclear	Yes	Yes	Yes	Unclear	Yes	Yes	Yes	No	Unclear
Singh et al. 2020	Yes	No	Yes	Unclear	Yes	Yes	Not applicable	Yes	Yes	Unclear
Tafti et al. 2020	Yes	No	Yes	Unclear	Yes	Yes	Not applicable	Yes	Yes	Unclear
Heidarpour et al. 2021	Yes	Yes	Unclear	Yes	Yes	Yes	Not applicable	Yes	Not applicable	Yes
Hookey et al. 2021	Yes	Yes	Unclear	Yes	Yes	Yes	Not applicable	Yes	Not applicable	Yes
Wu et al. 2022	Yes	Yes	Yes	Yes	Yes	Yes	Unclear	Yes	Not applicable	Yes

Q1: Were there clear criteria for inclusion in the case series? **Q2**: Was the condition measured in a standard, reliable way for all participants included in the case series? **Q3**: Were valid methods used for identification of the condition for all participants included in the case series? **Q4**: Did the case series have consecutive inclusion of participants? **Q5**: Did the case series have complete inclusion of participants? **Q6**: Was there clear reporting of the demographics of the participants in the study? **Q7**: Was there clear reporting of clinical information of the participants? **Q8**: Were the outcomes or follow-up results of cases clearly reported? **Q9**: Was there clear reporting of the presenting site(s)/clinic(s) demographic information? **Q10**: Was statistical analysis appropriate?

Table S4.3. Evaluation of observational studies using NOS.

First author/year of publication	SELECTION	COMPARABILITY	EXPOSURE/OUTCOME	Score
Albaba et al. 2021	****	*	*	6
Ali et al. 2021	***	*	*	5
Geng et al. 2021	****	*	**	7
Haroun et al. 2021	***	*	*	5
Martinot et al. 2021	****	*	**	7
Mohamed et al. 2021	***	*	**	6
Mokhtari et al. 2021	***	*	**	6
Zamoner et al. 2021	***	*	**	6
Khatib et al. 2022	****	*	*	6
Nashwan et al. 2023	****	*	**	7
Hashemi et al. 2024	****	*	*	6
Murt and Altiparmak 2024	***	*	*	5
Samardzic et al. 2024	****	*	*	6
